# Rapid detection of metastatic lymph nodes of colorectal cancer with a gamma-glutamyl transpeptidase-activatable fluorescence probe

**DOI:** 10.1038/s41598-018-36062-3

**Published:** 2018-12-12

**Authors:** Hidemasa Kubo, Kenjiro Hanaoka, Yugo Kuriki, Toru Komatsu, Tasuku Ueno, Ryosuke Kojima, Mako Kamiya, Yasutoshi Murayama, Eigo Otsuji, Yasuteru Urano

**Affiliations:** 1 Graduate School of Pharmaceutical Sciences, The University of Tokyo, 7-3-1 Hongo, Bunkyo-ku, Tokyo 113-0033 Japan; 2 Division of Digestive Surgery, Department of Surgery, Kyoto Prefectural University of Medicine, 465 Kajii-cho, Kamigyo-ku, Kyoto 602-8566 Japan; 3 Graduate School of Medicine, The University of Tokyo, 7-3-1 Hongo, Bunkyo-ku, Tokyo 113-0033 Japan; 4 Precursory Research for Embryonic Science and Technology (PRESTO) Investigator, Japan Science and Technology Agency (JST), 4-1-8 Honcho Kawaguchi, Saitama, 332-0012 Japan; 5 CREST (Japan) Agency for Medical Research and Development (AMED), 1-7-1 Otemachi, Chiyoda-ku, Tokyo 100-0004 Japan

## Abstract

Rapid diagnosis of metastatic lymph nodes (mLNs) of colorectal cancer (CRC) is desirable either intraoperatively or in resected fresh specimens. We have developed a series of activatable fluorescence probes for peptidase activities that are specifically upregulated in various tumors. We aimed to discover a target enzyme for detecting mLNs of CRC. Among our probes, we found that gGlu-HMRG, a gamma-glutamyl transpeptidase (GGT)-activatable fluorescence probe, could detect mLNs. This was unexpected, because we have previously reported that gGlu-HMRG could not detect primary CRC. We confirmed that the GGT activity of mLNs was high, whereas that of non-metastatic lymph nodes and CRC cell lines was low. We investigated the reason why GGT activity was upregulated in mLNs, and found that GGT was induced under conditions of hypoxia or low nutritional status. We utilized this feature to achieve rapid detection of mLNs with gGlu-HMRG. GGT appears to be a promising candidate enzyme for fluorescence imaging of mLNs.

## Introduction

Colorectal cancer (CRC) is one of the most common cancers worldwide^1^. Lymph node metastasis is often present in patients with advanced CRC, and is an important prognostic factor^2^. Thus, appropriate lymphadenectomy is critical during operation. The range of lymphadenectomy is usually decided based on preoperative diagnosis using computed tomography (CT), magnetic resonance imaging (MRI) and fluorodeoxyglucose-positron emission tomography (FDG-PET), but their sensitivity for diagnosing metastatic lymph nodes (mLNs) was reported to be only 55%, 66% and 29%, respectively^3,4^. Therefore, intraoperative confirmation can be important to ensure complete resection. This is usually done by histopathological analysis of frozen sections. However, only a few slides are generally examined, because the procedure is time-consuming, and so micrometastases may easily be missed^5–10^. Therefore, there is a need for rapid and accurate intraoperative diagnosis of mLN to improve the operative outcome.

Recently, fluorescence imaging has been investigated for fast intraoperative diagnosis. Indocyanine green (ICG) is used for sentinel lymph node imaging for CRC and other cancers^11–13^, but does not provide cancer-specific imaging. 5-Aminolevulinic acid (5-ALA) was reported to be useful for tumor imaging of CRC^14–16^. However, 5-ALA has to be administered orally prior to surgery, and there is a risk of skin sensitization^17^. On the other hand, we have recently developed a series of activatable fluorescence probes^18–20^ that can detect cancer cells based on their overexpression of a specific enzymatic activity, resulting in a high Tumor/Normal signal ratio. These fluorescence probes can visualize cancer cells within just a few minutes after application by topical spraying. We have already reported that gGlu-HMRG, a gamma-glutamyl transpeptidase (GGT)- activatable fluorescence probe, is available to detect ovary^18^, breast^21^ and lung^22^ cancers, and a dipeptidyl peptidase IV- activatable fluorescence probe could detect esophageal cancer^19^. However, a target enzyme for detecting mLNs of CRC has not yet been reported.

We previously found that gGlu-HMRG was not effective for detecting primary human CRC^23^. Nevertheless, we show here that mLNs of CRC can be rapidly detected with gGlu-HMRG. We further established that the reason for this is the induction of GGT under conditions of hypoxia and low nutritional status, resulting in high levels of GGT activity in mLNs.

## Results

### Rapid detection of metastatic lymph nodes of CRC

**Figure 1:**
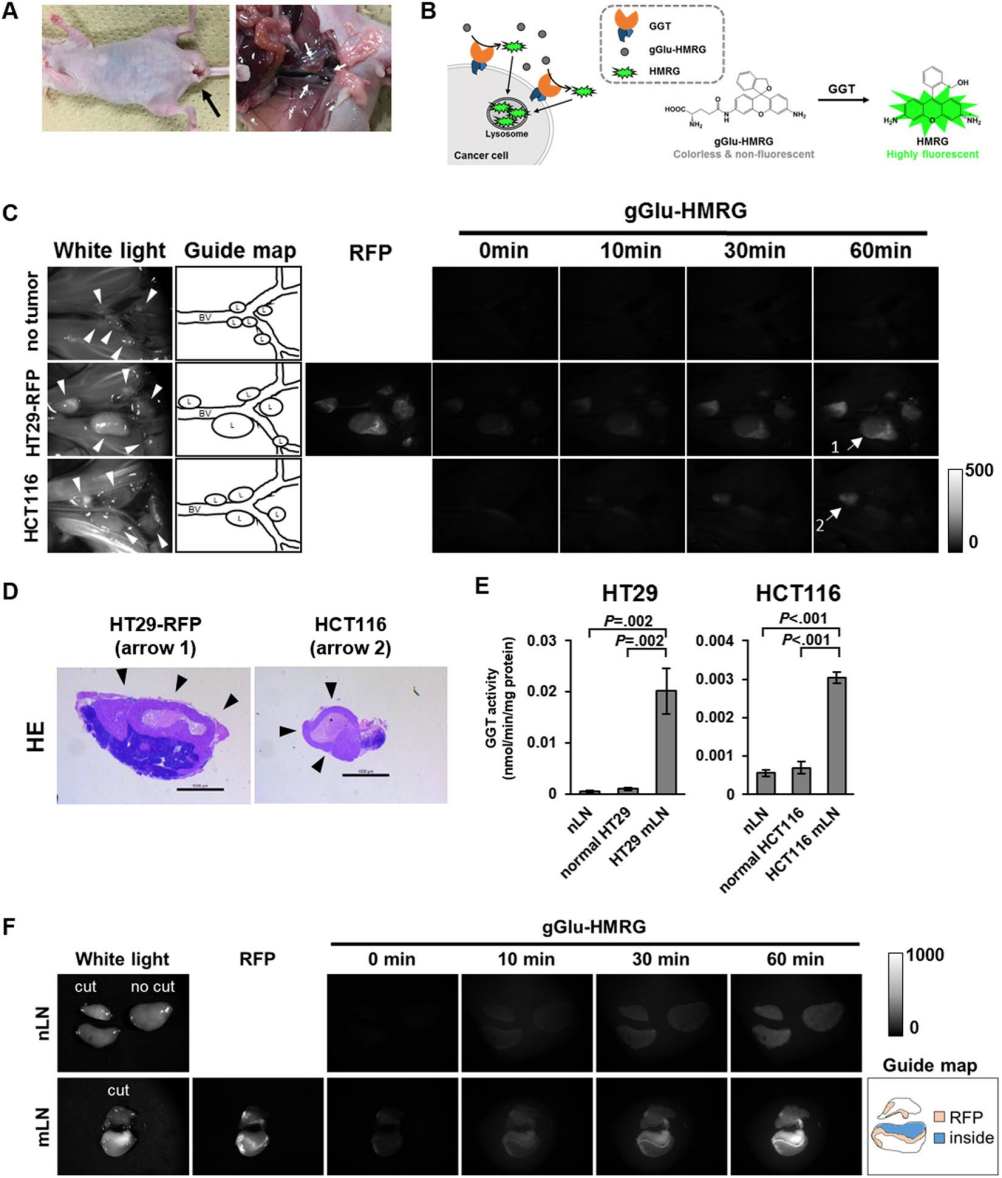
The GGT activity was upregulated in mLNs of CRC in model mouse. By utilizing this property, mLNs could be visualized with gGlu-HMRG *in vivo* and *ex vivo*. (**A**) Mouse model of para-aortic lymph node metastasis. The black arrow shows the primary tumor. White arrows show para-aortic lymph nodes. (**B**) Proposed mechanism of gGlu-HMRG activation and retention. gGlu-HMRG reacts with GGT in the plasma membrane of cells, generating the highly fluorescent product HMRG, which accumulates in lysosomes in the cells. (**C**,**D**) Lymph-node images of tumor-free mouse and the mouse model are shown. White light and RFP images were captured before application of gGlu-HMRG. Time-lapse images are shown after topical application of gGlu-HMRG. White arrowheads show lymph nodes. HE staining (arrows 1 and 2) shows that the lymph nodes detected by gGlu-HMRG are mLNs. Black arrowheads indicate metastatic lesions. BV: blood vessel, L: lymph node. Scale bar, 1000 μm. (**E**) GGT activity of whole tissue lysate of mLNs compared with normal cultured cells and nLNs (n = 3). Error bars represent SD. (**F**) *Ex vivo* imaging of nLN and mLN of HT29-RFP (cut surface) are shown. White light and RFP images were captured before application of gGlu-HMRG. Time-lapse images are shown after topical application of gGlu-HMRG. For the study of mLNs (**C** and **F**), we used a fluorescence stereoscopic microscope. For imaging of RFP, we used a G filter set (excitation filter 546/10 nm, barrier filter 590 nm long-pass). For imaging of gGlu-HMRG, we used a GFP3 filter set (excitation filter 470/40 nm, barrier filter 525/50 nm). mLNs: metastatic lymph nodes, nLNs: non-metastatic lymph nodes, GGT: gamma-glutamyl transpeptidase, HE: hematoxylin-eosin.

In the mouse model used here (HT29-RFP and HCT116 were injected into the posterior wall of the rectum), para-aortic lymph node metastasis occurred about six weeks after injection of cells (Fig. 1A). Among our activatable fluorescence probes for aminopeptidases and dipeptidyl peptidases, we found that gGlu-HMRG could visualize mLN *in vivo* (Fig. 1B–D, Supplementary Figure 1A,B). This was unexpected, because gGlu-HMRG could not successfully image primary human CRC^23^. We confirmed that the GGT activity of non-metastatic lymph nodes (nLNs) and cultured CRC cells was low, whereas the GGT activity of mLNs was high (Fig. 1E, Supplementary Figure 1C). We examined *ex vivo* imaging of nLN and mLN of HT29-RFP (cut surface) with gGlu-HMRG, and found that metastatic lesion including inside showed higher fluorescence than nLN (Fig. 1F, Supplementary Figure 1D).

### Imaging and assessment of GGT activity of the primary tumor

**Figure 2:**
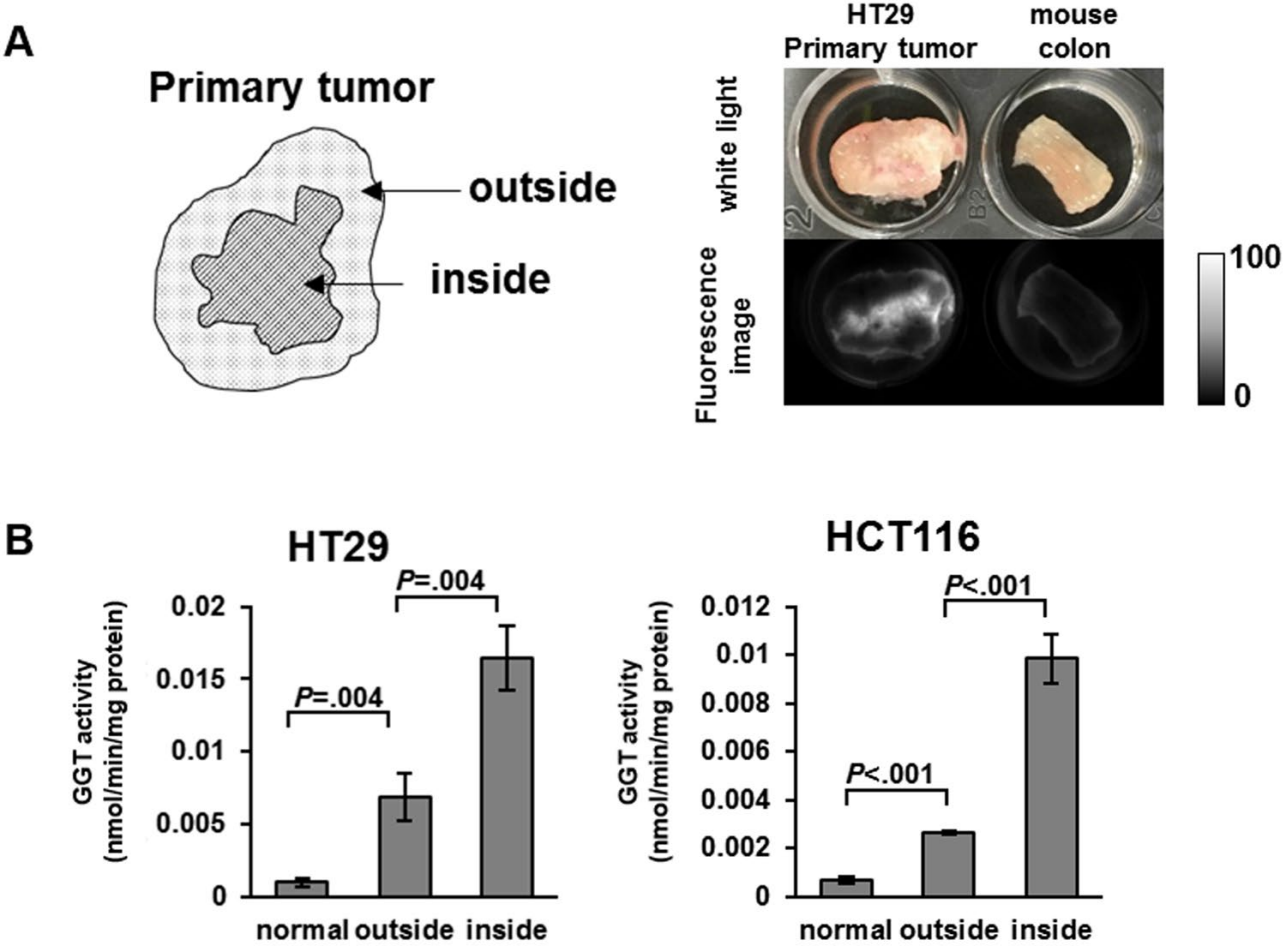
Study of the primary tumor in the mouse model. GGT activity of the inside of tumor was higher than the outside and normal cultured cells. (**A**) The locations ‘inside’ and ‘outside’ the tumor are shown. Comparison of HT29 primary tumor and mouse normal colon. White light and 540 nm fluorescence images, captured 30 min after application of gGlu-HMRG, are shown. (**B**) GGT activity of lysate of normal cultured cells, and the outside and inside of the tumor (n = 3). Error bars represent SD.

Next, we investigated the GGT activity of the primary tumor in this model mouse. After spraying gGlu-HMRG, we observed higher fluorescence in the inside (cut surface) of the primary tumor than on the outside, or in normal colon (Fig. 2A). The fluorescence was suppressed by a GGT inhibitor (Supplementary Figure 2A). GGT activity inside the tumor was higher than that of cultured cells or the outside surface of the tumor (Fig. 2B).

### Upregulation of GGT in CRC cell lines cultured under hypoxia or Met/Cys-free condition

**Figure 3:**
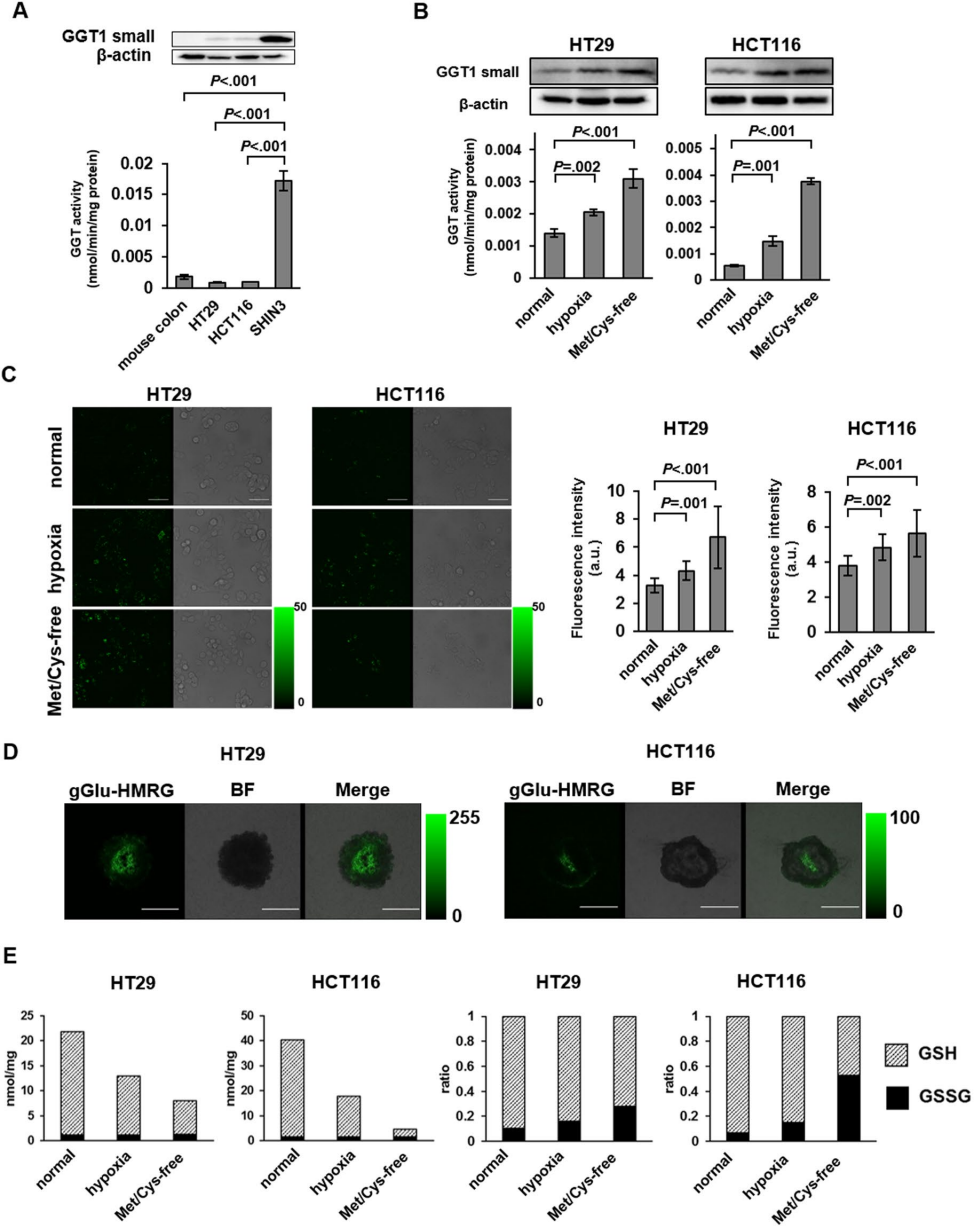
The GGT activity levels in colorectal cancer cell lines and mouse normal colon were lower than that of ovarian cancer cell line which is a positive control of GGT-expressing cell. The GGT activity and expression level of cell lines were upregulated in cells cultured under hypoxic and low nutritional conditions. In both hypoxic and low nutritional conditions, GSH decreased and GSSG increased, indicating that the cells were exposed to oxidative stress. (**A**) Comparison of GGT activity in mouse normal colon, HT29 cells, HCT116 cells, and ovarian cancer cell line SHIN3 as a positive control (n = 3). Error bars represent SD. Blots were cropped from different membrane divided after transfer. (**B**) Comparison of GGT activity and expression among CRC cell lines cultured under normal, hypoxic and Met/Cys-free conditions (n = 3). Error bars represent SD. In western blots, we detected GGT1 firstly, and then detected β-actin from the same membrane. (**C**) Live-cell fluorescence imaging of CRC cell lines cultured under normal, hypoxic and Met/Cys-free conditions with gGlu-HMRG. Scale bar, 50 μm. Average fluorescence intensity of ten cells selected at random are shown (n = 10). Error bars represent SD. (**D**) Fluorescence imaging of spheroids with gGlu-HMRG. Scale bar, 500 μm. (**E**) Measurement of GSH and GSSG in CRC cell lines cultured under normal, hypoxic and Met/Cys-free conditions. Absolute concentration was measured in lysate at 0.5 mg/ml protein concentration (n = 3). GSH: reduced glutathione, GSSG: oxidized glutathione.

To understand why GGT activity was upregulated in mLNs and inside the primary tumor, we compared the GGT activity of HT29 and HCT116 cells with that of SHIN3, an ovarian cancer cell line used as a positive control. The GGT activity levels in HT29 and HCT116 cells and in mouse normal colon were lower than that of SHIN3 (Fig. 3A, Supplementary Figure 3A). Live cell imaging with gGlu-HMRG confirmed that the fluorescence intensity of HT29 and HCT116 was lower than that of SHIN3 (Supplementary Figure 3B).

Based on these results, we hypothesized that GGT was induced by hypoxia and/or low nutritional status inside the tumor due to insufficient blood supply. Since GGT is related to cysteine homeostasis^24^, we investigated the effect of methionine-free, cystine-free (Met/Cys-free) conditions as a model of low nutritional status. Indeed, the GGT activity and expression level of cell lines were upregulated in cells cultured under Met/Cys-free conditions, compared to those under normal conditions (Fig. 3B, Supplementary Figure 3C). Live cell imaging and flow-cytometric analysis of cells cultured under hypoxic or Met/Cys-free conditions showed higher fluorescence as compared with cells cultured under normal conditions (Fig. 3C, Supplementary Figure 3D). We further confirmed that the increase of GGT1 under hypoxic or Met/Cys-free conditions was blocked by two specific siRNAs (Supplementary Figure 3E–H).

Next, we prepared spheroids as a three-dimensional tumor model; the concentrations of oxygen and nutrients are known to be low in the center of spheroids^25^. In live imaging of spheroids with gGlu-HMRG, higher fluorescence was indeed observed in the inside of spheroids than on the outside (Fig. 3D). Immunohistochemistry for GGT1 and CA9, a hypoxic marker, in formalin-fixed spheroids showed that GGT1 was expressed inside spheroids (Supplementary Figure 3I). Lysate of spheroids showed higher GGT expression and activity than lysate of monolayer-cultured cells (Supplementary Figure 3J,K). The upregulation of GGT1 in spheroids was blocked by two specific siRNAs (Supplementary Figure 3L,M).

The concentrations of reduced glutathione (GSH) and oxidized glutathione (GSSG) were examined in cells cultured under hypoxic and Met/Cys-free conditions. In both cases, GSH decreased and GSSG increased (Fig. 3E), indicating that the cells were exposed to oxidative stress^26^.

### Immunohistochemistry of primary tumor and mLNs

**Figure 4:**
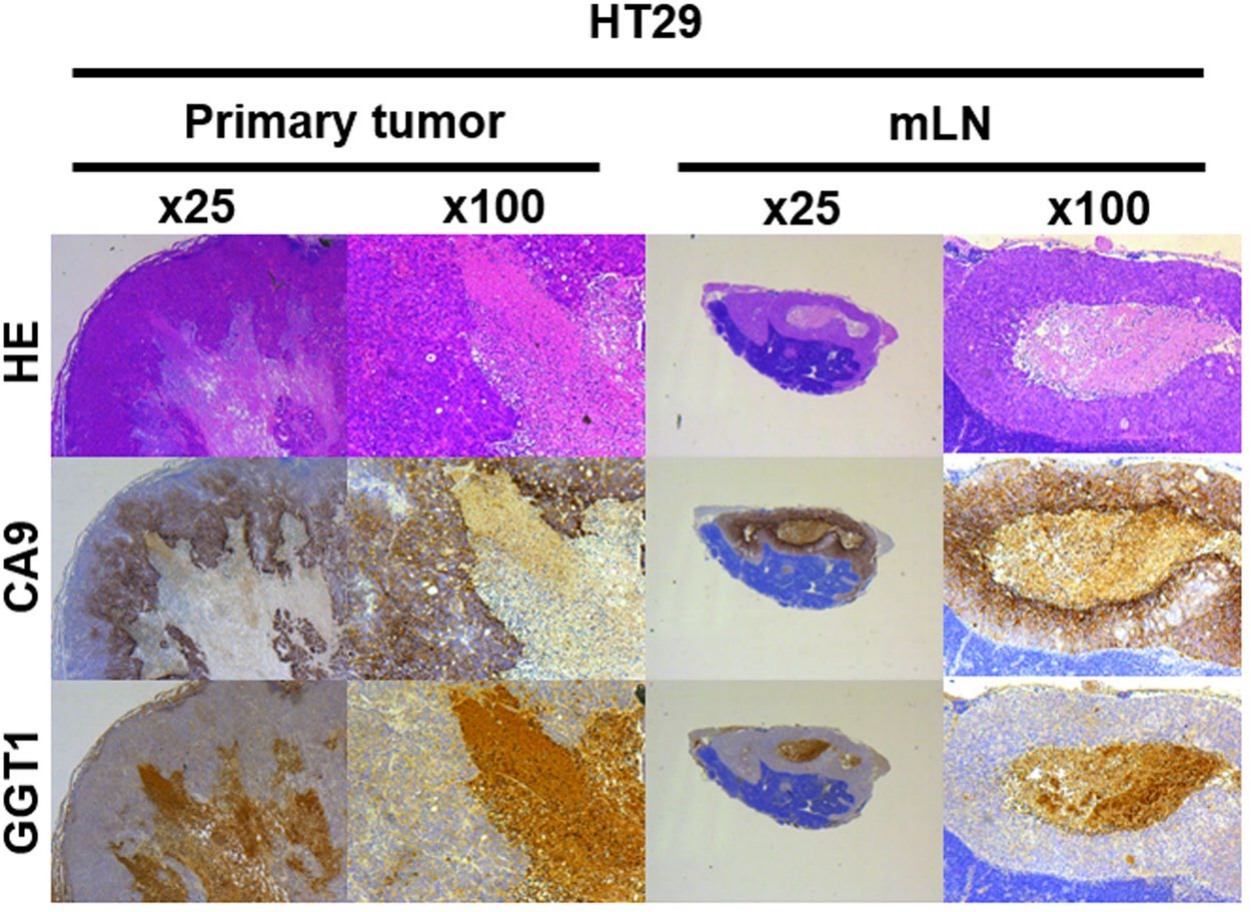
HE staining and immunohistochemistry (CA9 and GGT1) of fixed primary tumor and mLN specimens from orthotopic mouse model. CA9 and GGT1 were expressed inside the primary tumor and in mLNs. GGT1 was accumulated in the area of central necrosis. HE: hematoxylin-eosin.

Based on these results, we performed immunohistochemistry of GGT1 and CA9 for primary tumor and mLNs of the mouse model. GGT1 and CA9 were expressed inside the primary tumor and in mLNs. Especially, GGT1 was accumulated markedly in the area of central necrosis (Fig. 4, Supplementary Figure 4A). GSH was depleted inside the tumor (Supplementary Figure 4B).

### Oxygen concentration in lymph nodes and submucosa

**Figure 5:**
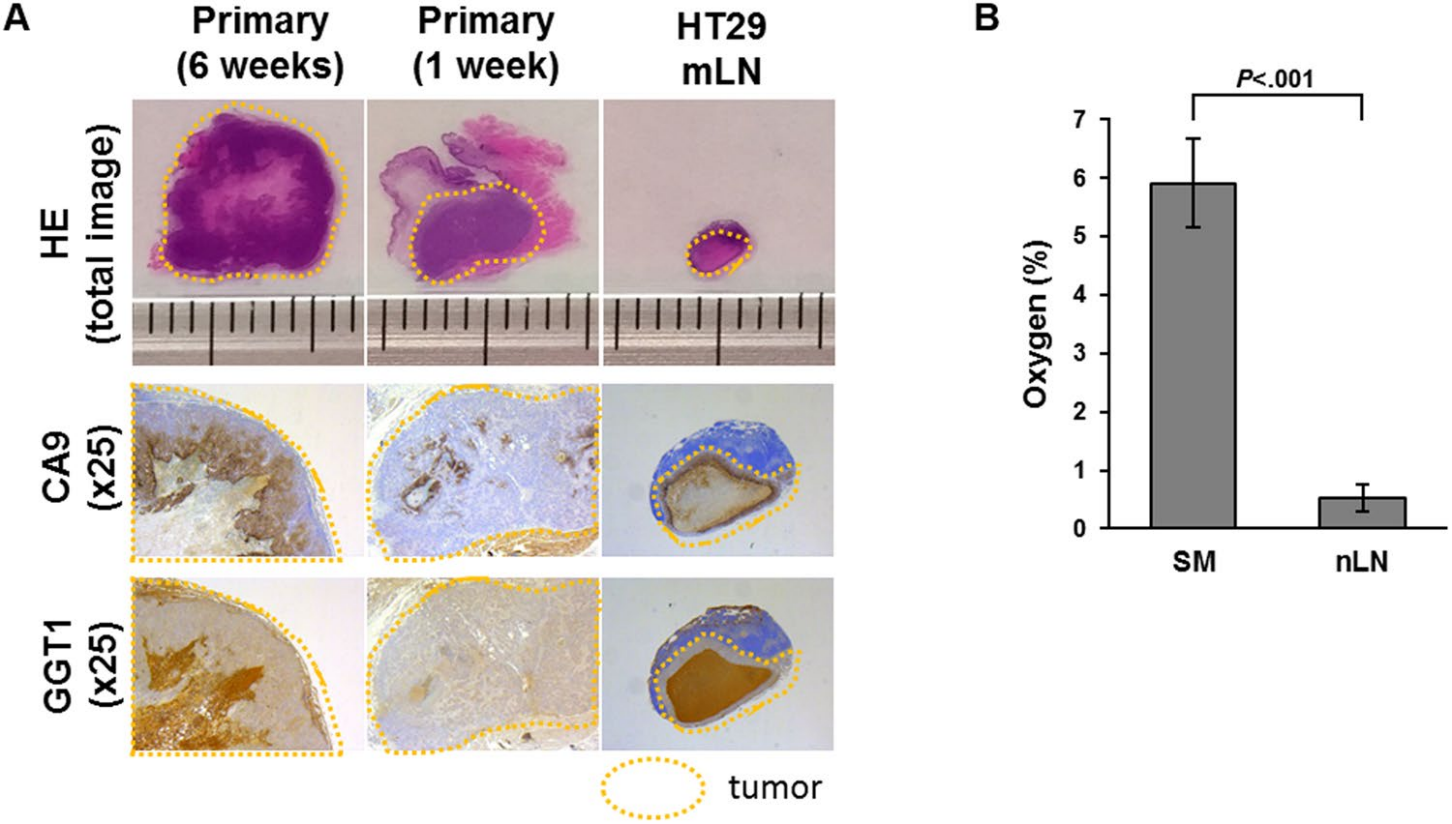
Comparison between primary tumor and mLNs of the mouse model. Expression of CA9 and GGT1 in mLNs was higher than that in the primary tumor at 1week after injection of cells. (**A**) CA9 and GGT1 expression of HT29 mLNs compared to primary tumor at 6 weeks or 1 week after injection of cells. (**B**) Tissue oxygen concentration (%) of rectal submucosa (SM) and nLNs (n = 3). Error bars represent SD. mNs: metastatic lymph nodes, nLNs: non metastatic lymph nodes.

We next examined the oxygen concentration in HT29 primary tumor one week after injection and in mLNs. The inside of mLN exhibited central necrosis, and expression of CA9 and GGT1 was higher than that in the primary tumor, despite the small size of the mLNs (Fig. 5A), suggesting that blood supply did not depend on size. Interestingly, the oxygen concentration of nLNs was also lower than that of the rectal submucosa (Fig. 5B, Supplementary Figure 5). These results suggest that the lymph node environment favors induction of GGT, possibly because the oxygen concentration is lower than that in submucosa.

### Clinical case presentation

To examine whether the above findings are relevant to human CRC, we investigated twelve human CRC specimens (Case 1–12). Case 10–12 had received pre-operative chemoradiotherapy.

**Figure 6:**
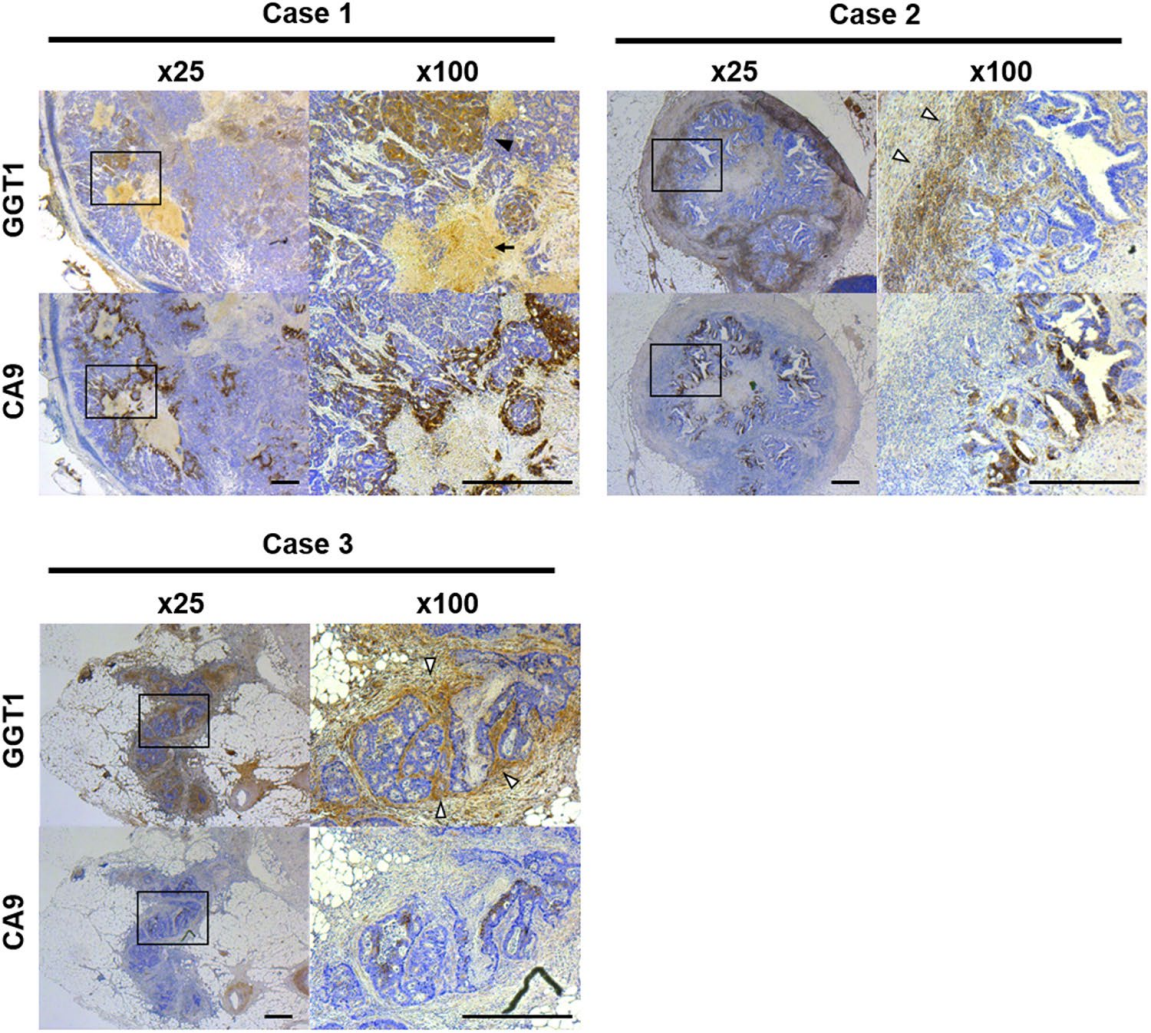
Representative human colorectal cancer specimen. Immunohistochemistry of CA9 and GGT1 in mLNs. In these cases, both CA9 and GGT1 were expressed, suggesting that GGT1 expression is related to hypoxia in mLNs. Scale bar, 500 μm. Black arrowheads: GGT of cancer cells, white arrowheads: GGT surrounding cancer cells, arrow: accumulation of GGT. mLNs: metastatic lymph nodes, GGT: gamma-glutamyl transpeptidase.

The mLNs of Cases 1–6, 10 and 11 expressed GGT1, while Cases 7 and 12 showed only weak expression and Cases 8 and 9 expressed little (Fig. 6, Supplementary Figure 6). In Cases 1–3, both CA9 and GGT1 were expressed, suggesting that GGT1 expression is related to hypoxia in mLNs. In Case 1, 5 and 10, GGT1 was accumulated in the necrotic lesions of mLNs. In the primary tumor of Cases 1, 4 and 7, the expression patterns of GGT1 and CA9 were similar, while the expression patterns of GGT1 and CA9 were different in Cases 2, 3, 5, 6, and 8–12 (Supplementary Figure 7).

Some cases expressed GGT1 in cancer cells, and others expressed GGT1 not only in cancer cells, but also in the interstitial spaces around them. This may suggest that GGT1 was derived from both cancer cells and stromal cells. Overall, our findings indicate that gGlu-HMRG might to be applicable for imaging mLNs in the clinical context.

## Discussion

In this work, we show that GGT is induced under conditions of hypoxia and low nutritional status, and we utilized this characteristic to achieve rapid detection of mLNs with gGlu-HMRG. Our results suggest that GGT is a promising candidate as a target enzyme for rapid detection of mLNs of CRC.

CT, MRI and FDG-PET are important diagnostic techniques for cancer^3,4^, but despite advances in preoperative imaging with these modalities, the surgical margin rate has not changed significantly for several decades^27,28^. Intraoperative CT and MRI are used in neurosurgery^29–31^, but are costly, complex and inconvenient. Therefore, activatable fluorescence probes are expected to be more practical for fast and straightforward intraoperative diagnosis^27^. However, as these probes require enzymatic activation, it is necessary to identify a target enzyme that is specifically overexpressed in each cancer. Here, we found for the first time that GGT is a candidate for imaging mLNs in patients with CRC.

GGT plays an important role of glutathione metabolism, and is known to be related to cancer progression, invasion and drug resistance^32,33^. Although the GGT activity of CRC cell lines is low, we found here that hypoxia and low nutritional status (Met/Cys-free conditions) induced oxidative stress^26^, leading to induction of GGT. This is consistent with previous reports that GGT is induced by oxidative stress^34–38^.

We confirmed that GGT highly expressed in mLNs and the inside of the primary tumor in a mouse model of CRC. Therefore, although we previously reported that the GGT probe gGlu-HMRG could not visualize primary human CRC^23^, mLNs could be visualized with gGlu-HMRG. We think this is because GGT expression is induced in cancer cells located in the lymph node where the oxygen concentration is lower than that in submucosa.

The immunohistochemical study revealed that, at least in some cases, such as Cases 2, 3, 11, GGT was expressed in the interstitial space surrounding cancer cells rather than in the cancer cells. It is reported that GGT derived from macrophage lineage cells is accumulated in atherosclerotic plaques^39^. So, it is possible that GGT in interstitial space is derived from not only cancer cells, but also stromal cells. Further studies of this issue with larger numbers of cases are needed. Nevertheless, our results indicate that most human mLN samples examined expressed GGT. Therefore, it appears that gGlu-HMRG might be effective for rapid detection of mLNs *ex vivo*. There are some limitations for applying to *in vivo* imaging. Human lymph nodes are usually located in adipose tissue, so topical spraying might not suitable for *in vivo* imaging, and fluorescence wavelength of HMRG is not sufficient for deep observation. However, our study indicate that GGT is a promising biomarker for detecting mLNs. We are planning further fluorescence imaging studies of fresh human tumor samples to confirm the diagnostic value of gGlu-HMRG. Furthermore, we will develop novel photoacoustic probe targeting GGT to overcome limitations mentioned above for *in vivo* imaging in the future.

## Methods

### Mouse model of lymph node metastasis

All procedures were carried out in compliance with the usual requirements for animal experiments, and were approved by the University of Tokyo. BALB/c Ajcl-nu/nu female mice were purchased from CLEA Japan, Inc. Under isoflurane anesthesia (Wako, Japan), the anterior wall of rectum was cut, and a suspension of 1 × 10^6^ tumor cells (see below) in a mixture of equal volumes of phosphate-buffered saline (PBS) and Matrigel (Corning, MA, USA) was injected into the posterior wall of the rectum. Fluorescence imaging was performed six or more weeks after this procedure.

### *In vivo* and *ex vivo* fluorescence imaging

For the study of mLNs, we used a fluorescence stereoscopic microscope (Leica, MZFLIII). For imaging of HMRG, we used a GFP3 filter set (excitation filter 470/40 nm, barrier filter 525/50 nm). For imaging of RFP, we used a G filter set (excitation filter 546/10 nm, barrier filter 590 nm long-pass).

For primary tumor imaging, mice were sacrificed and primary tumors were excised and cut. Fluorescence images of the outside and the cut surface were obtained with a Maestro *In-Vivo* imaging system (Cri Inc.). The blue-filter setting (excitation, 445 to 490 nm; emission, 515 nm long-pass) was used for detection of HMRG. The tunable filter was automatically stepped in 10-nm increments from 500 to 800 nm while the camera sequentially captured images. GGsTop (Wako, Japan) was used as an inhibitor of GGT. We exported the 540 nm fluorescence images using Maestro software, and calculated the fluorescence intensity of regions of interest (ROI) using ImageJ software (NIH).

50 μM gGlu-HMRG was used for imaging of mLNs and primary tumor.

### GGT activity assay

GGT activity was measured with an EnVision multilabel plate reader (Perkin Elmer) in 384-well black plates with 5 μL/well lysate (1 mg/mL protein concentration) and 15 μL/well fluorescence probe solution (1.33 μM in PBS containing DMSO as a co-solvent, final: 1 μM). HMRG was used as the positive-control fluorophore and gGlu-HMRG as the fluorescence probe. Fluorescence intensity was measured every min for 120 min (FITC filter; ex/em 485/535), and the results of gGlu-HMRG assay were normalized to those of 1 μM of HMRG assay performed at the same time. The GGT activity was calculated as follows:

$$\begin{array}{ccc}{\rm{A}}{\rm{c}}{\rm{t}}{\rm{i}}{\rm{v}}{\rm{i}}{\rm{t}}{\rm{y}} & = & ({\rm{F}}{\rm{l}}{\rm{u}}{\rm{o}}{\rm{r}}{\rm{e}}{\rm{s}}{\rm{c}}{\rm{e}}{\rm{n}}{\rm{c}}{\rm{e}}\,{\rm{i}}{\rm{n}}{\rm{c}}{\rm{r}}{\rm{e}}{\rm{a}}{\rm{s}}{\rm{e}}\,{\rm{r}}{\rm{a}}{\rm{t}}{\rm{e}})/({\rm{F}}{\rm{l}}{\rm{u}}{\rm{o}}{\rm{r}}{\rm{e}}{\rm{s}}{\rm{c}}{\rm{e}}{\rm{n}}{\rm{c}}{\rm{e}}\,{\rm{i}}{\rm{n}}{\rm{t}}{\rm{e}}{\rm{n}}{\rm{s}}{\rm{i}}{\rm{t}}{\rm{y}}\,{\rm{o}}{\rm{f}}\,{\rm{H}}{\rm{M}}{\rm{R}}{\rm{G}}\,{\rm{i}}{\rm{n}}\,{\rm{l}}{\rm{y}}{\rm{s}}{\rm{a}}{\rm{t}}{\rm{e}}\\  &  & -\,{\rm{F}}{\rm{l}}{\rm{u}}{\rm{o}}{\rm{r}}{\rm{e}}{\rm{s}}{\rm{c}}{\rm{e}}{\rm{n}}{\rm{c}}{\rm{e}}\,{\rm{i}}{\rm{n}}{\rm{t}}{\rm{e}}{\rm{n}}{\rm{s}}{\rm{i}}{\rm{t}}{\rm{y}}\,{\rm{o}}{\rm{f}}\,{\rm{g}}{\rm{G}}{\rm{l}}{\rm{u}}{\textstyle \text{-}}{\rm{H}}{\rm{M}}{\rm{R}}{\rm{G}}\,{\rm{j}}{\rm{u}}{\rm{s}}{\rm{t}}\,{\rm{a}}{\rm{f}}{\rm{t}}{\rm{e}}{\rm{r}}\,{\rm{a}}{\rm{d}}{\rm{d}}{\rm{i}}{\rm{t}}{\rm{i}}{\rm{o}}{\rm{n}}\,{\rm{o}}{\rm{f}}\,{\rm{l}}{\rm{y}}{\rm{s}}{\rm{a}}{\rm{t}}{\rm{e}})\\  &  & /({\rm{p}}{\rm{r}}{\rm{o}}{\rm{t}}{\rm{e}}{\rm{i}}{\rm{n}}\,{\rm{c}}{\rm{o}}{\rm{n}}{\rm{c}}{\rm{e}}{\rm{n}}{\rm{t}}{\rm{r}}{\rm{a}}{\rm{t}}{\rm{i}}{\rm{o}}{\rm{n}}).\end{array}$$

### Cell lines and culture

Two human colorectal cancer cell lines and one human ovarian cancer cell line were used in this study: HT29 (RIKEN BioResource Center, Japan); HCT116 (American Type Culture Collection); SHIN3 (provided by S. Imai, Nara, Japan). HT29-RFP cells transfected with DsRed2 as RFP from coral were purchased from AntiCancer Japan. HT29 and HCT116 cells were grown in McCoy’s 5a medium (Gibco), and SHIN3 cells were grown in RPMI 1640 medium (Gibco) at 37 °C in 5% CO_2_. Both media contained 10% fetal bovine serum (Biowest), penicillin (100 U/mL) and streptomycin (100 μg/mL). Hypoxic cultures were prepared by incubation in an atmosphere containing under 1% O_2_ (Sanyo, MCO-5M) for 1 day. Cultures under Met/Cys-free conditions were prepared in RPMI 1640 (Sigma R7513; without L-methionine, L-cystine, L-glutamine), containing L-glutamine (0.3 g/L), 10% dialyzed FBS (Gibco), penicillin (100 U/mL) and streptomycin (100 μg/mL) in 5% CO_2_ for 1 day.

Spheroid cultures were prepared in 96-well U-bottomed plates (Corning) coated with poly 2-hydroxyethyl methacrylate (Sigma). Cells were suspended in culture medium containing 2.5% Matrigel basement membrane matrix (Corning, MA, USA) at a concentration of 50,000 cells/mL, and 100 μL of the suspension was dispensed into each well (5,000 cells/well). The plate was centrifuged and incubated for 4 days to prepare spheroids.

### siRNA knockdown of GGT1

siRNA transfection was performed by applying 10 nM (final concentration) siRNA-1 or siRNA-2 targeting GGT1 or 10 nM nonsense control to each colorectal cancer cell line. Lipofectamine RNAiMAX Transfection Reagent (Invitrogen, CA, USA) was used according to the manufacturer’s protocol.

HT29 was incubated for 3 days, and HCT116 was incubated for 2 days under hypoxic or Met/Cys-free conditions. Spheroids of all cell lines were incubated for 4 days.

GGT1 siRNA-1 sense: 5′rCrArArCrArGrCrArCrCrArCrArCrGrArArArArGrCr

antisense: 5′UUUUrCrGUrGUrGrGUrGrGUrGUUrGUrA

Control siRNA-1 sense: 5′GUrArCrCrGrCrArCrGUrCrAUUrCrGUrAUrC

antisense: 5′UrArCrGrArAUrGrArCrGUrGrCrGrGUrArCrGU

siRNA-2 was purchased from Santa Cruz: GGT1 siRNA (h) (sc-35473) and Control siRNA-A (sc-37007).

### Live-cell fluorescence imaging

1 × 10^5^ cells of HT29, HCT116 and SHIN3 were plated on a cover glass-bottomed culture well and incubated at 37 °C under 5% CO_2_ for 1 day. The cells were washed with Hanks’ Balanced Salt solution (HBSS, Gibco) twice, then 1 μM gGlu-HMRG in HBSS was added, and incubation was continued for 15 or 60 min. Fluorescence images were captured with a Leica Application Suit Advanced Fluorescence (LAS-AF) microscope with TCS SP5 and a 63x objective lens (excitation/emission, 488/505-600). Bright field (BF) images were also acquired.

For comparison of the fluorescence intensities of CRC cell lines cultured under normal, hypoxic and Met/Cys-free conditions, we performed fluorescence imaging. 1 × 10^5^ cells from each cell line were plated on a cover glass-bottomed culture well. Incubation conditions were as follows: normal cells: 37 °C, 5% CO_2_ for 1 day; hypoxic cells: 37 °C, 5% CO_2_, 1% O_2_ for 1 day; Met/Cys-free cells: Met/Cys-free medium at 37 °C, 5% CO_2_ for 1 day. For fluorescence imaging, the same procedure described above was used. Incubation time after addition of gGlu-HMRG solution was 15 min.

The fluorescence intensity of cells was calculated using ImageJ software (NIH).

### Flow-cytometric analysis

GGT activity of HT29 and HCT116 cell lines cultured under normal condition, hypoxic and Met/Cys-free conditions was examined with gGlu-HMRG by flow cytometry. Cells cultured under each condition for 1 day were washed with HBSS and collected with a cell scraper. The cells were treated with 1 μM gGlu-HMRG and 1 μM ethidium homodimer 1 (Wako, Japan) in HBSS at 37 °C for 15 min, passed through a cell strainer (FALCON, 35 μm mesh), and analyzed in a BD LSR II Flow Cytometer (BD Biosciences). Data were analyzed with FlowJo software. Fluorescence of gGlu-HMRG was detected with an FITC filter after exclusion of dead cells based on ethidium homodimer 1 fluorescence.

### Preparation of cell lysate

To evaluate enzymatic activity of GGT, we prepared cell and tissue lysates. For preparation of cell lysates, cultured cells were washed with PBS, then CelLytic M (SIGMA, MO, USA) was added, and the cells were homogenized in an ultrasonic homogenizer. The homogenate was centrifuged for 10 min at 14,000 × g in 4 °C. For preparation of tissue lysates, tissues were suspended in CelLytic M, crushed with scissors, homogenized in an ultrasonic homogenizer, and centrifuged as described above. The supernatant was collected and the protein concentration was quantified by the BCA Protein Assay Kit (Thermo Scientific, IL, USA).

### Western blotting

To evaluate protein expression of GGT, we performed western blotting. Before loading on the gel, the lysate was deglycosylated with PNGase F (New England Biolabs, P0704, MA, USA) and sample buffer (β-ME Sample Treatment for Tris SDS, DCB, Japan) was added. The mixture was boiled at 105 °C for 3 min. Polyacrylamide gel electrophoresis was performed and bands were transferred to PVDF membrane. Primary antibodies were as follows: anti GGT1 for detecting GGT1 small subunit (M01, H00002678, Abnova, Taiwan) and anti β-actin (C4, sc-47778, SantaCruz, USA). The secondary antibody was anti-mouse IgG (GE Healthcare, NA931). We used ECL Prime Western Blotting System (GE Healthcare, RPN2232, USA) or Westar Supernova (CYANAGEN, XLS3, 0100, Italy) as a chemiluminescent reagent. Images were captured with an ImageQuant LAS 4000 mini (GE Healthcare).

### Determination of GSSG and GSH

A GSSG/GSH Quantification Kit (Dojindo Molecular Technologies, Inc. Japan) was used for evaluating GSSG and GSH concentrations of cells and tissues at 0.5 mg/mL protein concentration and in 0.5% sulfosalicylic acid lysate according to the manufacturer’s instructions.

### Measurement of tissue oxygen concentration

Oxygen concentrations in axillary lymph nodes and submucosa of rectum of BALB/c Ajcl nu/nu female mice were measured with a Microx TX3 oxygen-sensitive electrode (PreSens, Germany).

### Immunohistochemistry

Paraffin-embedded sections were deparafiinized by soaking in a xylene bath three times and in an ethanol bath twice. For CA9 antigen retrieval, the slides were soaked in 2 N HCl for 15 min at room temperature. For GGT1 antigen retrieval, the slides were soaked in Tris-EDTA (pH 9.0) for 30 min at 95 °C and cooled to room temperature for 30 min. The slides were then incubated in 3% H_2_O_2_/H_2_O for 20 min at room temperature to quench endogenous peroxidase activity, and incubated with primary antibody against CA9 (NB100-417, NOVUS, rabbit polyclonal, USA) or GGT1 (M01, H00002678, Abnova, mouse monoclonal, Taiwan) for 90 min at room temperature. For mouse tissue slides, Blocking Reagents A and B (TaKaRa POD Conjugate Set Anti Mouse, For Mouse Tissue, Japan) were used before and after applying primary antibody to quench endogenous immunoglobulin. Secondary antibody (TaKaRa POD conjugate anti Rabbit for CA9, anti Mouse for GGT1, Japan) was applied for 30 min at room temperature. 3,3′-Diaminobenzidine (DAB) was used as a chromogen for 5 min, and Mayer’s hematoxylin (Wako, Japan) was applied for 3 min as a counterstain. The sections were washed, dehydrated and then enclosed in mounting medium (Entellan new, Merck, Germany).

### Clinical specimens

Clinical specimens were obtained from colorectal cancer patients by surgery and were diagnosed as positive for lymph node metastasis at the University Hospital of Kyoto Prefectural University of Medicine (KPUM). All procedures were conducted in accordance with the Declaration of Helsinki, and were approved by the Institutional Review Boards of KPUM (approval number ERB-C-1000) and the University of Tokyo (approval number 29–15). Informed consent was obtained from all patients.

### Statistical analysis

Statistical analysis was performed with ystat 2008 software. The two-tailed unpaired *t*-test was used to compare GGT activity, fluorescence intensity of cells and oxygen concentration. Values of *P* < 0.05 were considered to be statistically significant.

## Electronic supplementary material

Supplementary information

## Data Availability

The datasets of the current study are available from the corresponding author on reasonable request.
