# Supplementary material for: Rapid detection of metastatic lymph nodes of colorectal cancer with a gamma-glutamyl transpeptidase-activatable fluorescence probe
Source: Sci Rep. 2018 Dec 12;8:17781. doi: 10.1038/s41598-018-36062-3 (PMC6290796; doi:10.1038/s41598-018-36062-3)
Supplement: Supplementary file 1 — Supplementary information [file 41598_2018_36062_MOESM1_ESM.pdf]

Supplementary information

Rapid detection of metastatic lymph nodes of colorectal cancer with a gamma-glutamyl transpeptidase-activatable fluorescence probe

Hidemasa Kubo[1,2], Kenjiro Hanaoka[1], Yugo Kuriki[1], Toru Komatsu[1], Tasuku Ueno[1], Ryosuke Kojima[3,4], Mako Kamiya[3,4], Yasutoshi Murayama[2], Eigo Otsuji[2], Yasuteru Urano[1,3,5,*]

[1]Graduate School of Pharmaceutical Sciences, The University of Tokyo, 7-3-1 Hongo, Bunkyo-ku, Tokyo 113-0033, Japan

[2]Division of Digestive Surgery, Department of Surgery, Kyoto Prefectural University of Medicine, 465 Kajii-cho, Kamigyo-ku, Kyoto, 602-8566, Japan

[3]Graduate School of Medicine, The University of Tokyo, 7-3-1 Hongo, Bunkyo-ku, Tokyo 113-0033, Japan

[4]Precursory Research for Embryonic Science and Technology (PRESTO) Investigator, Japan Science and Technology Agency (JST), 4-1-8 Honcho Kawaguchi, Saitama 332-0012, Japan

[5]CREST (Japan) Agency for Medical Research and Development (AMED), 1-7-1 Otemachi, Chiyoda-ku, Tokyo 100-0004, Japan

***Correspondence to:**

Yasuteru Urano, Graduate School of Pharmaceutical Sciences, The University of Tokyo, 7-3-1 Hongo, Bunkyo-ku, Tokyo 113-0033, Japan.

Phone: +81-3-5841-3601

E-mail: uranokun@m.u-tokyo.ac.jp

Supplementary Figures

Supplementary Figure 1

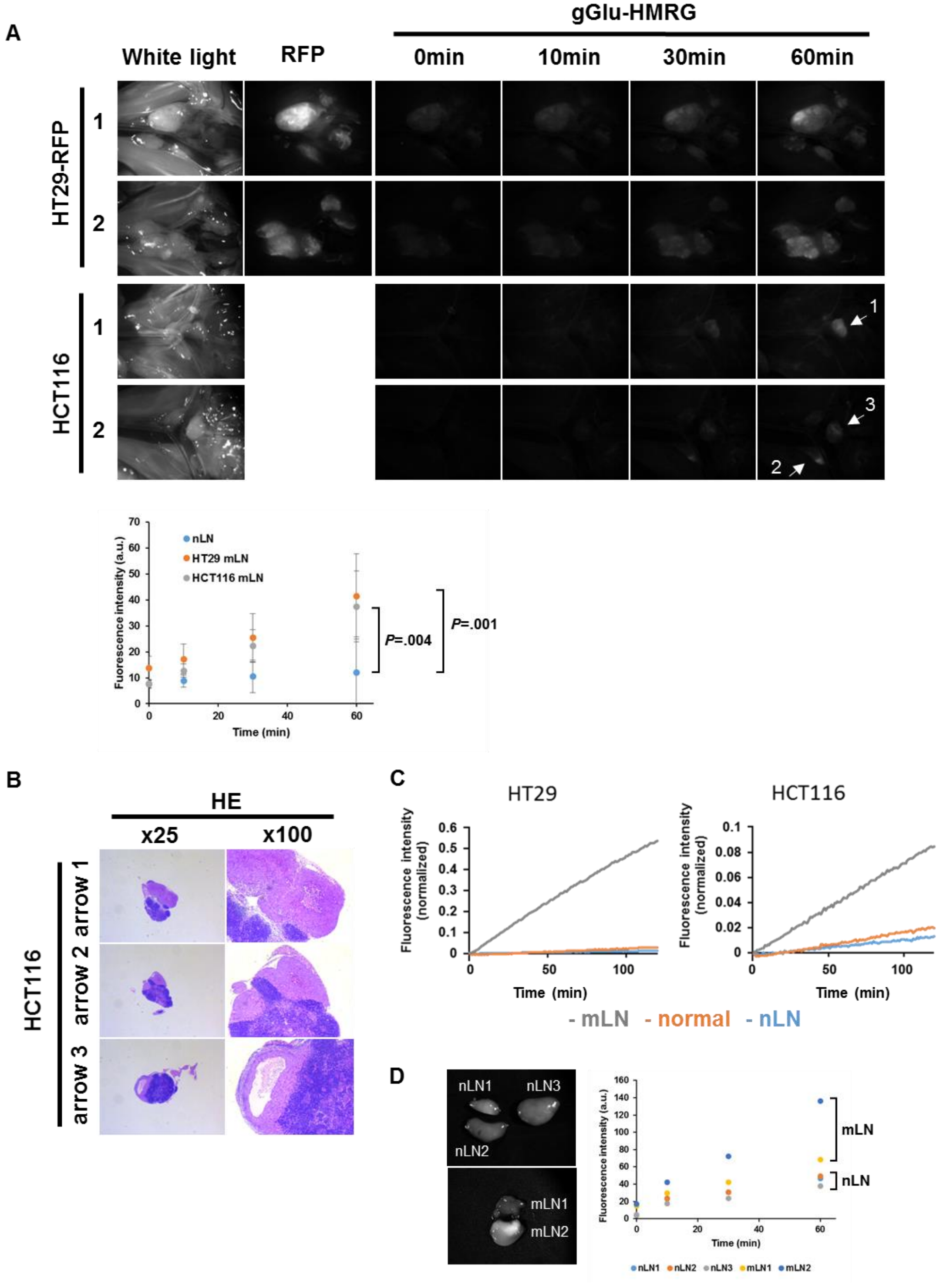

gGlu-HMRG can visualize mLNs of CRC in vivo. GGT activity is upregulated in mLNs. **A)** In lymph nodes of HT29-RFP, fluorescence of RFP almost matched the fluorescence of gGlu-HMRG. Some lymph nodes of HCT116 showed fluorescence of gGlu-HMRG. Graph shows time course of fluorescence intensity of nLN (n=5), mLN of HT29 (n=12) and HCT116 (n=4). Error bars represent SD. **B)** HE staining of three gGlu-HMRG fluorescence-positive lymph nodes of HCT116 mice. **C)** Time course of normalized fluorescence intensity of nLN, normal cultured cells, and mLNs with gGlu-HRMG. **D)** Time course of fluorescence intensity of nLN and mLN ex vivo after spraying gGlu-HMRG.

Supplementary Figure 2

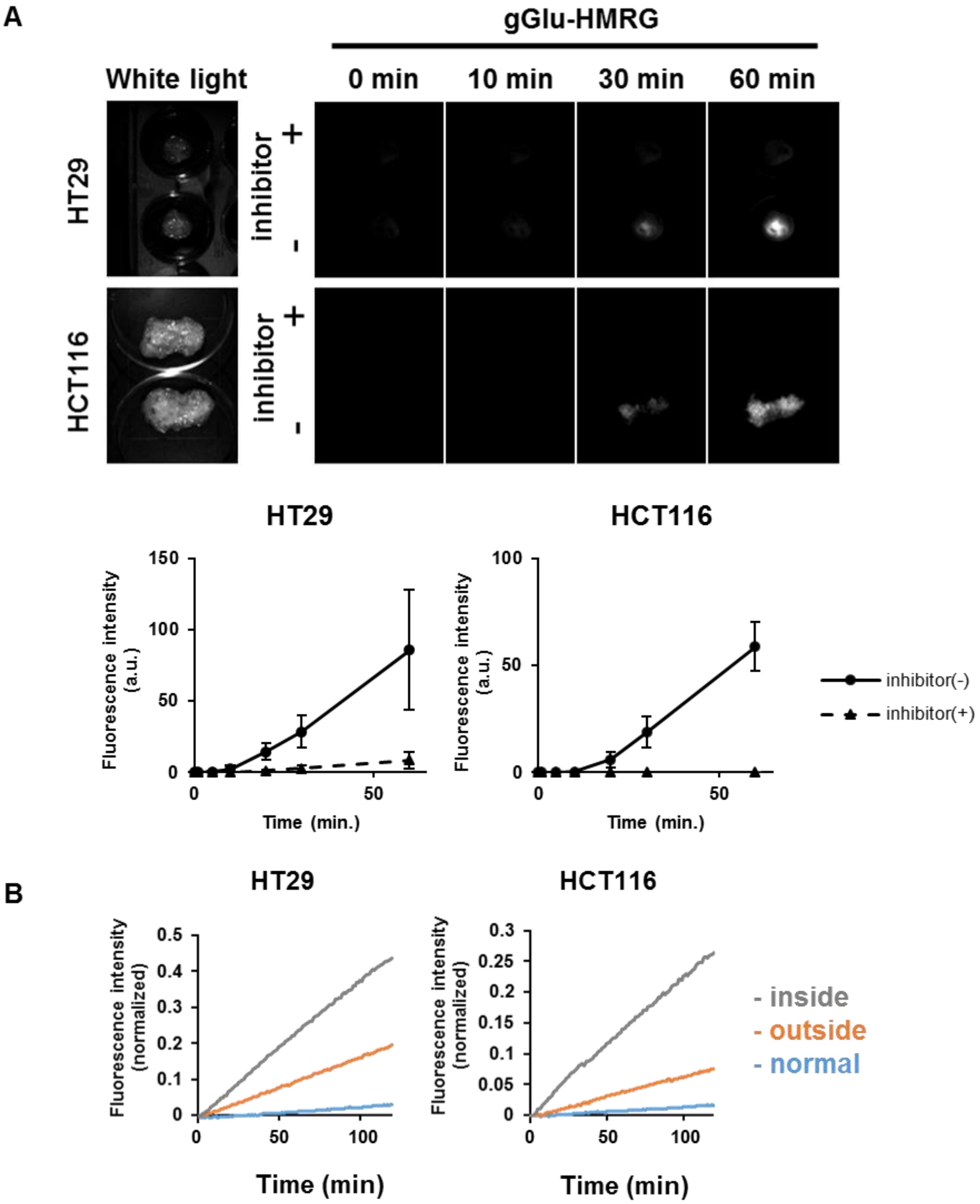

GGT activity of the inside of primary tumor is higher than the outside. **A)** Ex vivo imaging of the cut surface of a primary tumor after spraying gGlu-HMRG with or without GGT inhibitor (n=3). Error bars represent SD. **B)** Time course of normalized fluorescence intensity of normal cell line, and outside and inside of the primary tumor with gGlu-HMRG.

Supplementary Figure 3

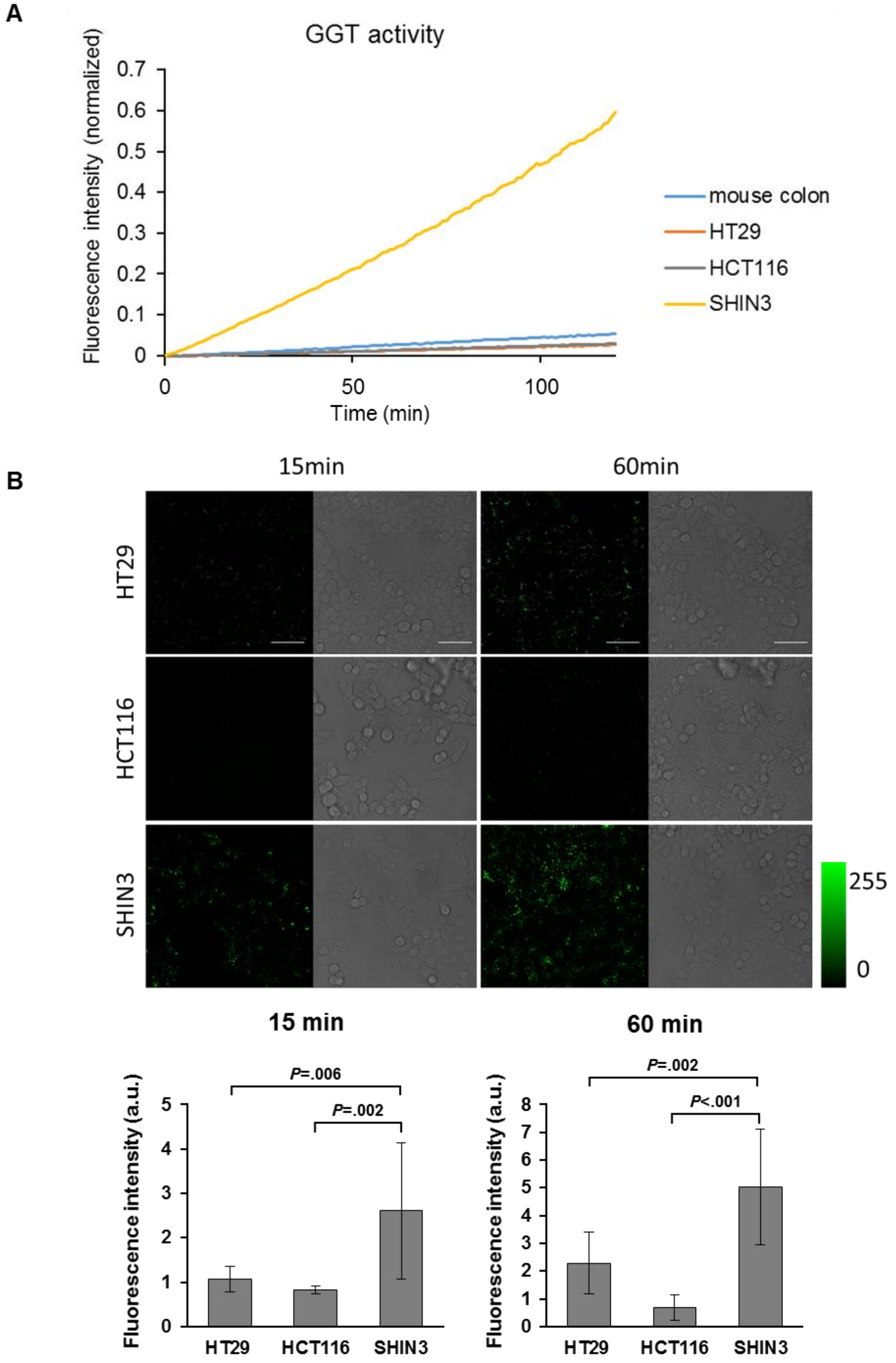

C

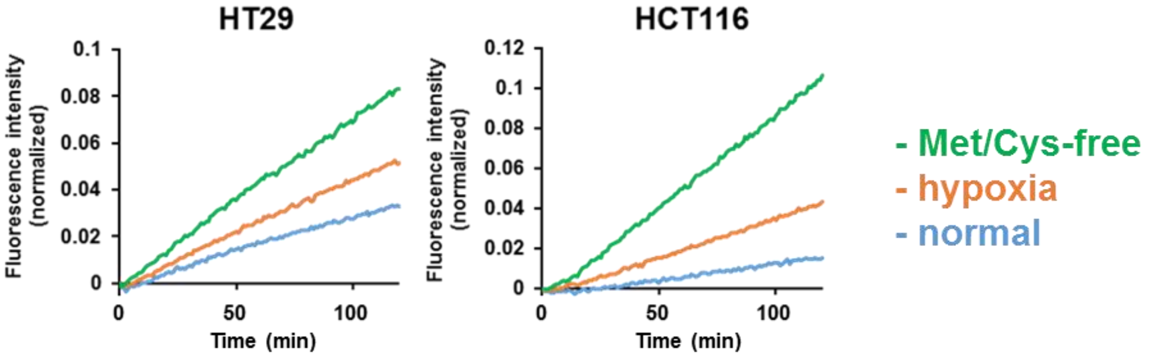
HT29
HCT116
Fluorescence intensity (normalized)
Time (min)
- Met/Cys-free
- hypoxia
- normal

D

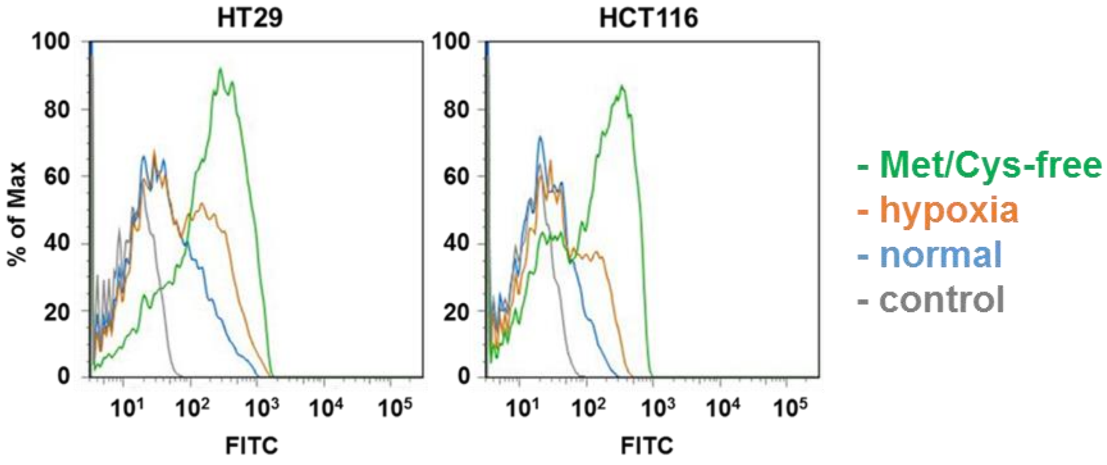
HT29
HCT116
% of Max
FITC
- Met/Cys-free
- hypoxia
- normal
- control

E

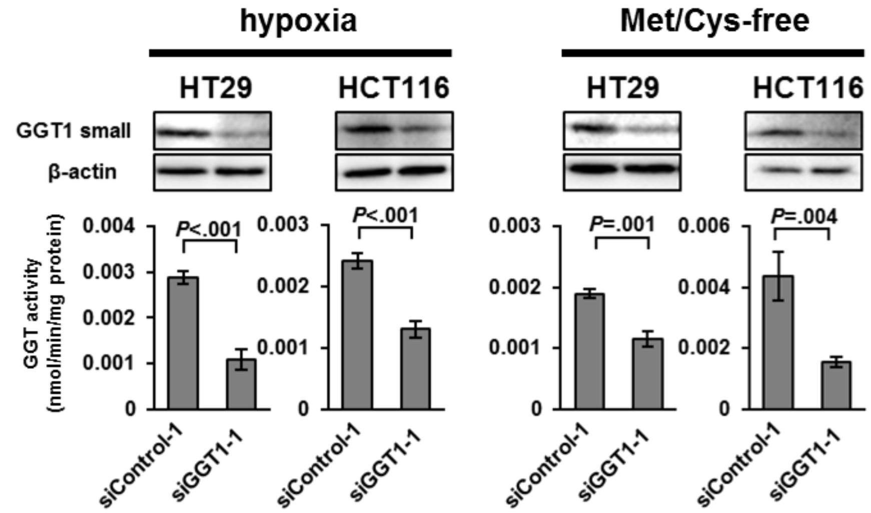

hypoxia
Met/Cys-free
HT29
HCT116
HT29
HCT116
GGT1 small
β-actin
GGT activity
(nmol/min/mg protein)
P<.001
P<.001
P=.001
P=.004
0.004
0.003
0.002
0.001
0
0.003
0.002
0.001
0
0.003
0.002
0.001
0
0.006
0.004
0.002
0
siControl-1
siGGT1-1

F

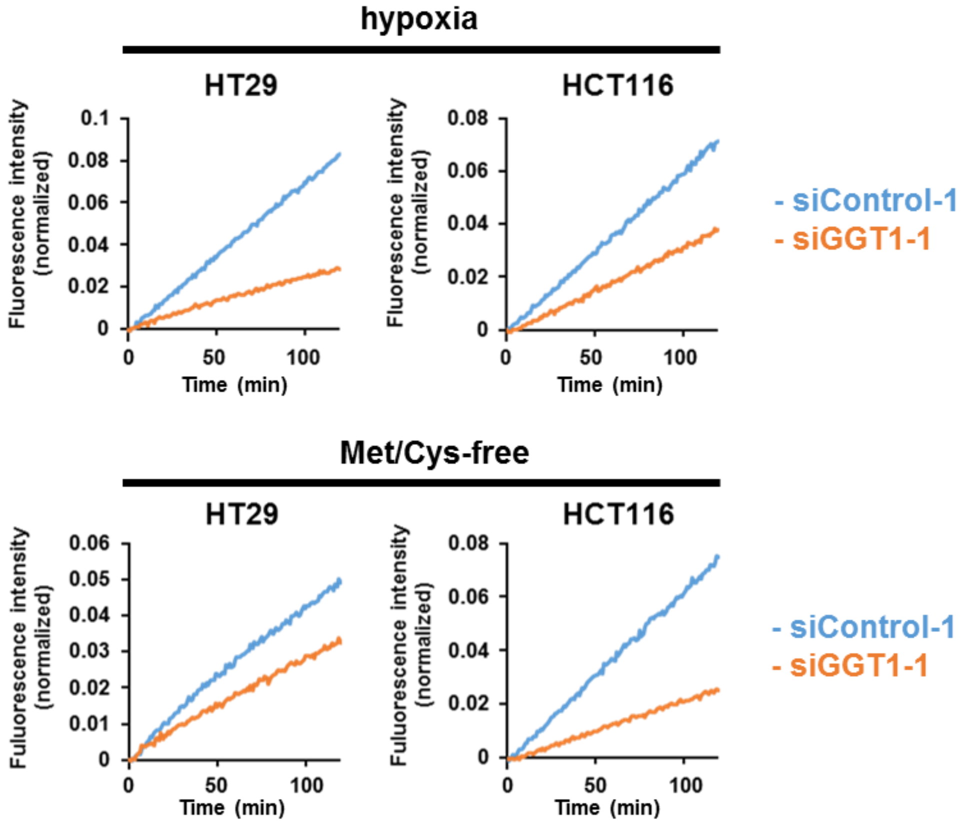

hypoxia
HT29
HCT116
Fluorescence intensity
(normalized)
0.1
0.08
0.06
0.04
0.02
0
0.08
0.06
0.04
0.02
0
0
50
100
Time (min)
- siControl-1
- siGGT1-1
Met/Cys-free
HT29
HCT116
Fuluorescence intensity
(normalized)
0.06
0.05
0.04
0.03
0.02
0.01
0
0.08
0.06
0.04
0.02
0
0
50
100
Time (min)
- siControl-1
- siGGT1-1

G

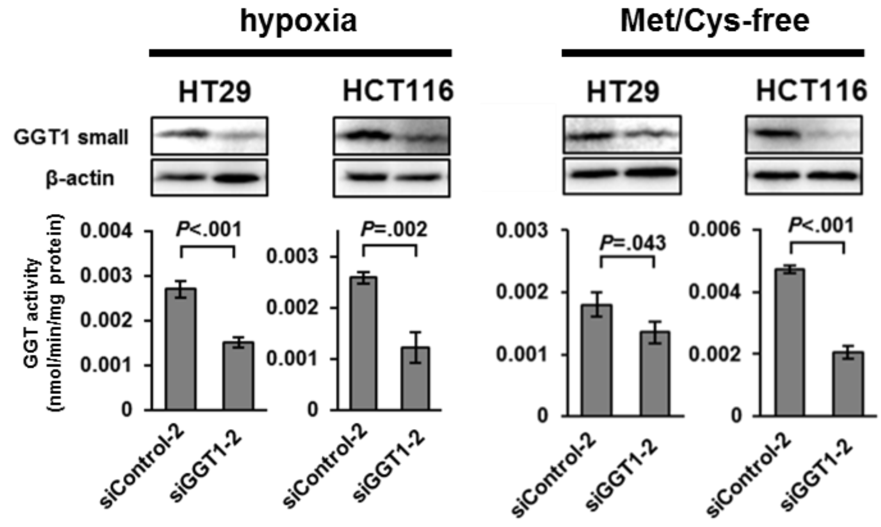
hypoxia
Met/Cys-free
HT29
HCT116
HT29
HCT116
GGT1 small
β-actin
GGT activity (nmol/min/mg protein)
P<.001
P=.002
P=.043
P<.001
0.004
0.003
0.002
0.001
0
0.006
siControl-2
siGGT1-2

H

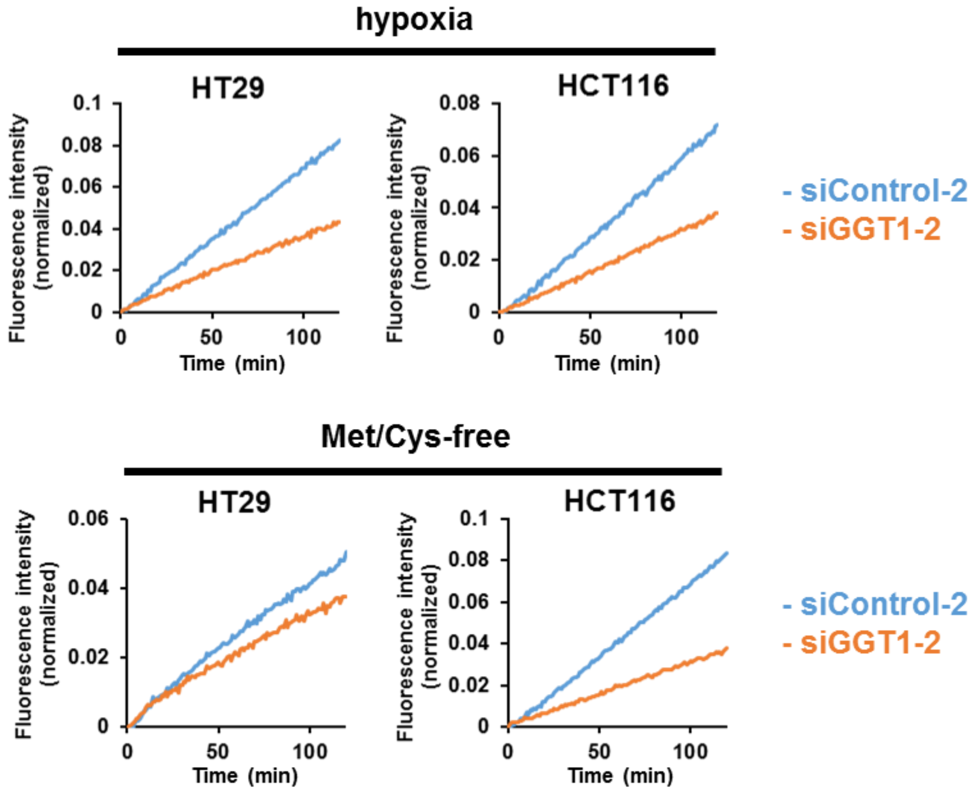
hypoxia
HT29
HCT116
Fluorescence intensity (normalized)
Time (min)
0.1
0.08
0.06
0.04
0.02
0
50
100
- siControl-2
- siGGT1-2
Met/Cys-free
HT29
HCT116
Fluorescence intensity (normalized)
Time (min)
- siControl-2
- siGGT1-2

I

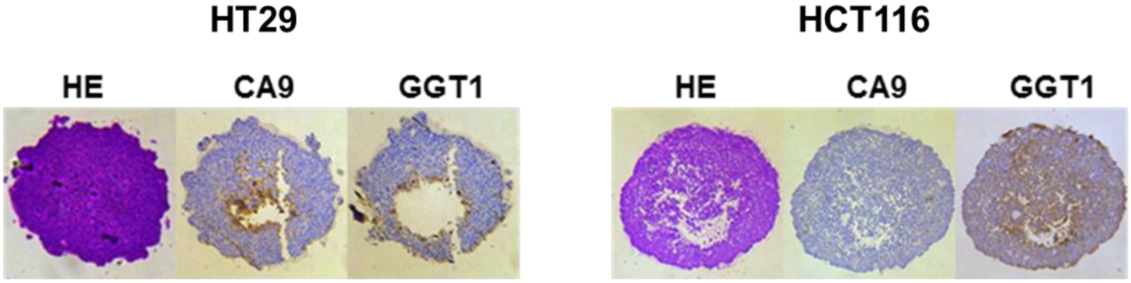
HT29
HE
CA9
GGT1
HCT116
HE
CA9
GGT1

J

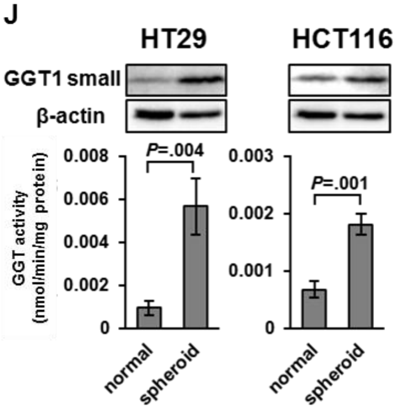
HT29
HCT116
GGT1 small
β-actin
GGT activity
(nmol/min/mg protein)
0.008
0.006
0.004
0.002
0
P=.004
normal
spheroid
0.003
0.002
0.001
0
P=.001
normal
spheroid

K

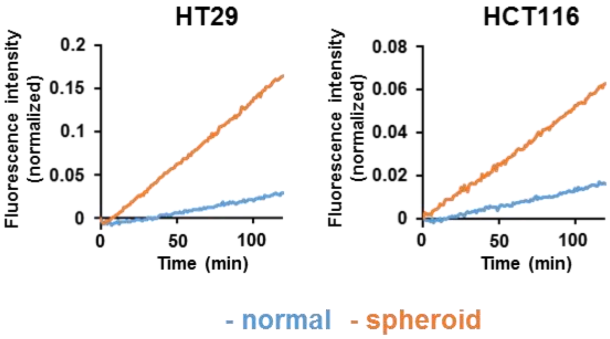
HT29
Fluorescence intensity
(normalized)
0.2
0.15
0.1
0.05
0
0
50
100
Time (min)
HCT116
Fluorescence intensity
(normalized)
0.08
0.06
0.04
0.02
0
0
50
100
Time (min)
- normal
- spheroid

L

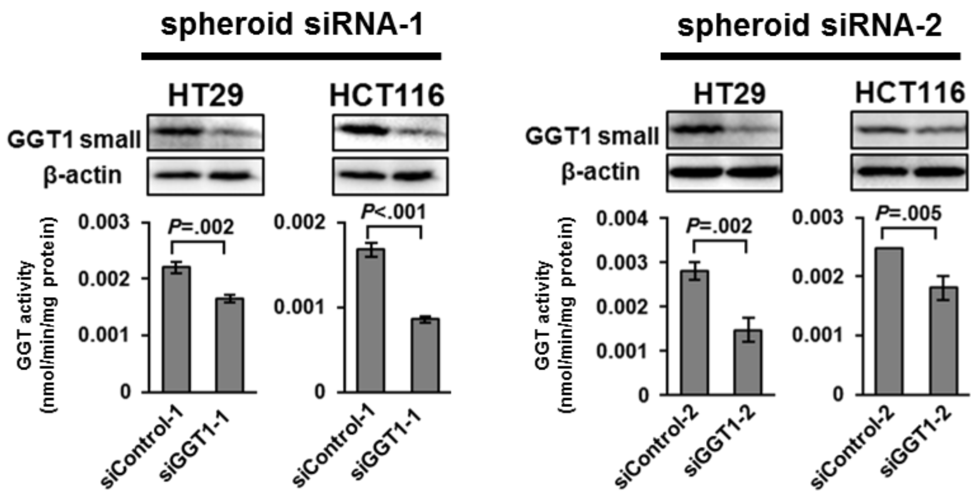
spheroid siRNA-1
spheroid siRNA-2
HT29
HCT116
HT29
HCT116
GGT1 small
β-actin
GGT1 small
β-actin
GGT activity (nmol/min/mg protein)
P=.002
P<.001
P=.002
P=.005
0.003
0.002
0.001
0
0.002
0.001
0
0.004
0.003
0.002
0.001
0
0.003
0.002
0.001
0
siControl-1
siGGT1-1
siControl-2
siGGT1-2

M

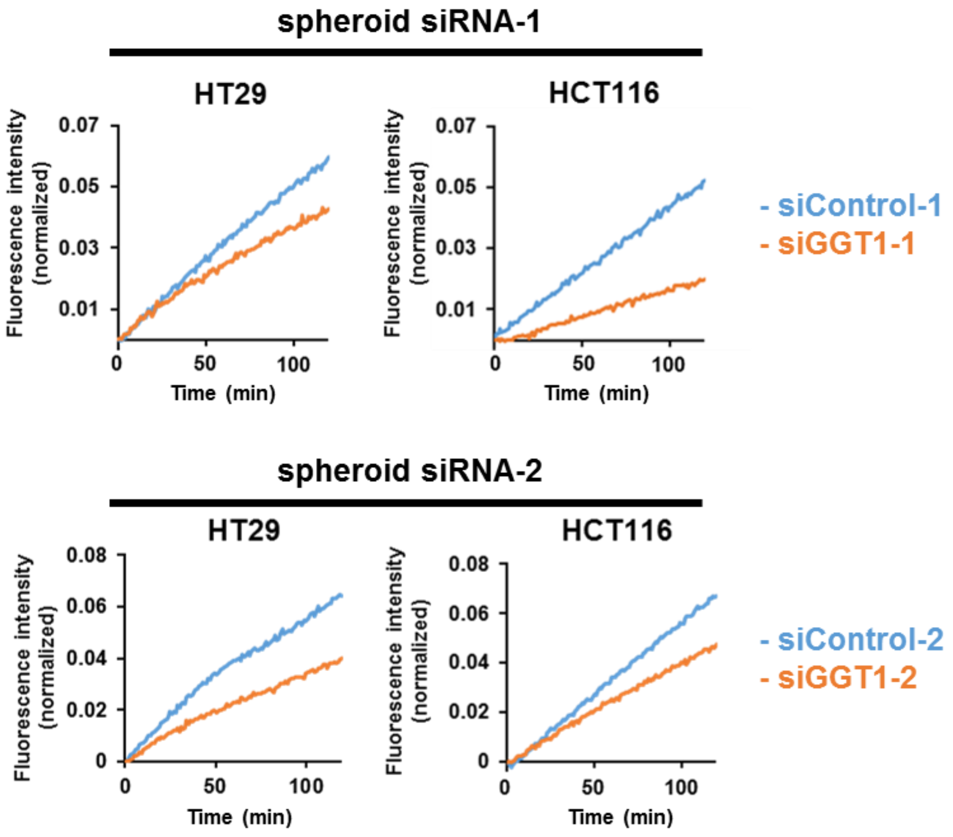
spheroid siRNA-1
HT29
HCT116
Fluorescence intensity (normalized)
0.07
0.05
0.03
0.01
0
50
100
Time (min)
- siControl-1
- siGGT1-1
spheroid siRNA-2
HT29
HCT116
0.08
0.06
0.04
0.02
0
- siControl-2
- siGGT1-2

N

<Figure 3A>

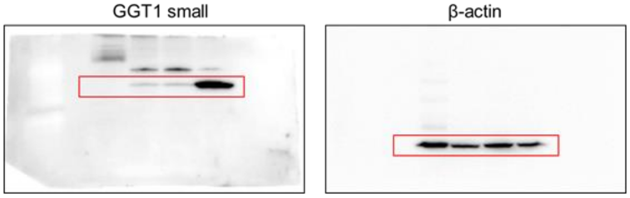

<Figure 3B>

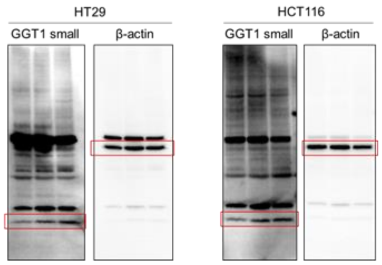

<Supplementary Figure 3E>

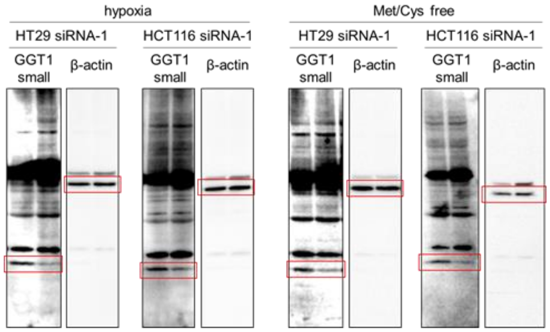

<Supplementary Figure 3G>

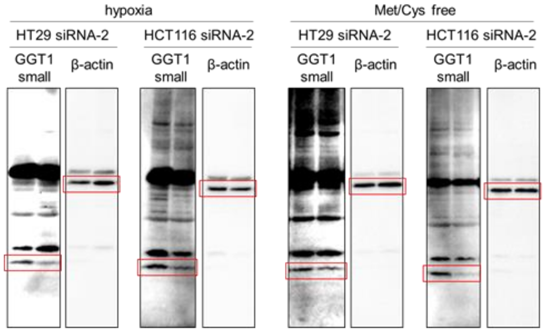

<Supplementary Figure 3J>

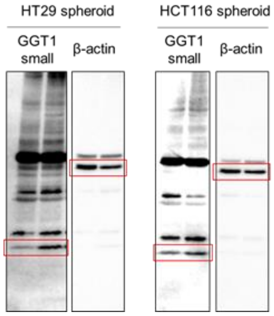

<Supplementary Figure 3L>

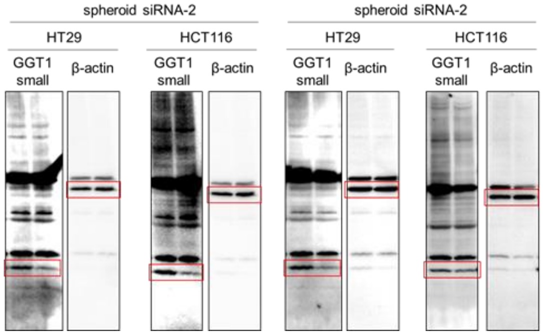

The GGT activity levels in colorectal cancer cell lines and mouse normal colon were lower than that of ovarian cancer cell line which is a positive control of GGT-expressing cell. Hypoxia and Met/Cys-free conditions induced GGT. GGT activity and expression levels were also upregulated in the inside of spheroids than the outside. **A)** GGT activity of

mouse normal colon, HT29, HCT116 and SHIN3. Graph shows the time course of normalized fluorescence intensity. **B)** Live-cell fluorescence imaging of HT29, HCT116 and SHIN3 incubated for 15 min and 60 min after applying gGlu-HMRG solution. Average fluorescence intensity of ten cells selected randomly in live-cell fluorescence imaging (n=10). Scale bars, 50 μm. Error bars represent SD. **C)** Time course of normalized fluorescence intensity of HT29 and HCT116 cultured under normal, hypoxic and Met/Cys-free conditions. **D)** Flow-cytometric analysis of HT29 and HCT116 cultured under normal, hypoxic and Met/Cys-free conditions with gGlu-HMRG. **E,F)** siRNA-1 knockdown of GGT1 in HT29 and HCT116 cultured under hypoxic and Met/Cys-free conditions was performed, and GGT1 expression, activity and the time course of normalized fluorescence intensity with gGlu-HMRG were measured (n=3). Error bars represent SD. **G,H)** siRNA-2 knockdown of GGT1 in HT29 and HCT116 cultured under hypoxia and Met/Cys-free conditions was performed, and GGT1 expression, activity and the time course of normalized fluorescence intensity with gGlu-HMRG were measured (n=3). Error bars represent SD. **I**) HE and immunohistochemistry (CA9 and GGT1) of spheroids. **J,K**) GGT activity, expression and the time course of normalized fluorescence intensity with gGlu-HMRG of spheroids compared to those of normal cultured cells (n=3). Error bars represent SD. **L,M**) siRNA-1, -2 knockdown of GGT1 in HT29 and HCT116 spheroids was performed, and GGT1 expression, activity and the time course of normalized fluorescence intensity with gGlu-HMRG were measured (n=3). Error bars represent SD. **N**) Full-length western blots. Red squares show the cropped bands. Firstly we detected GGT1, and then detected β-actin from the same membrane.

Supplementary Figure 4

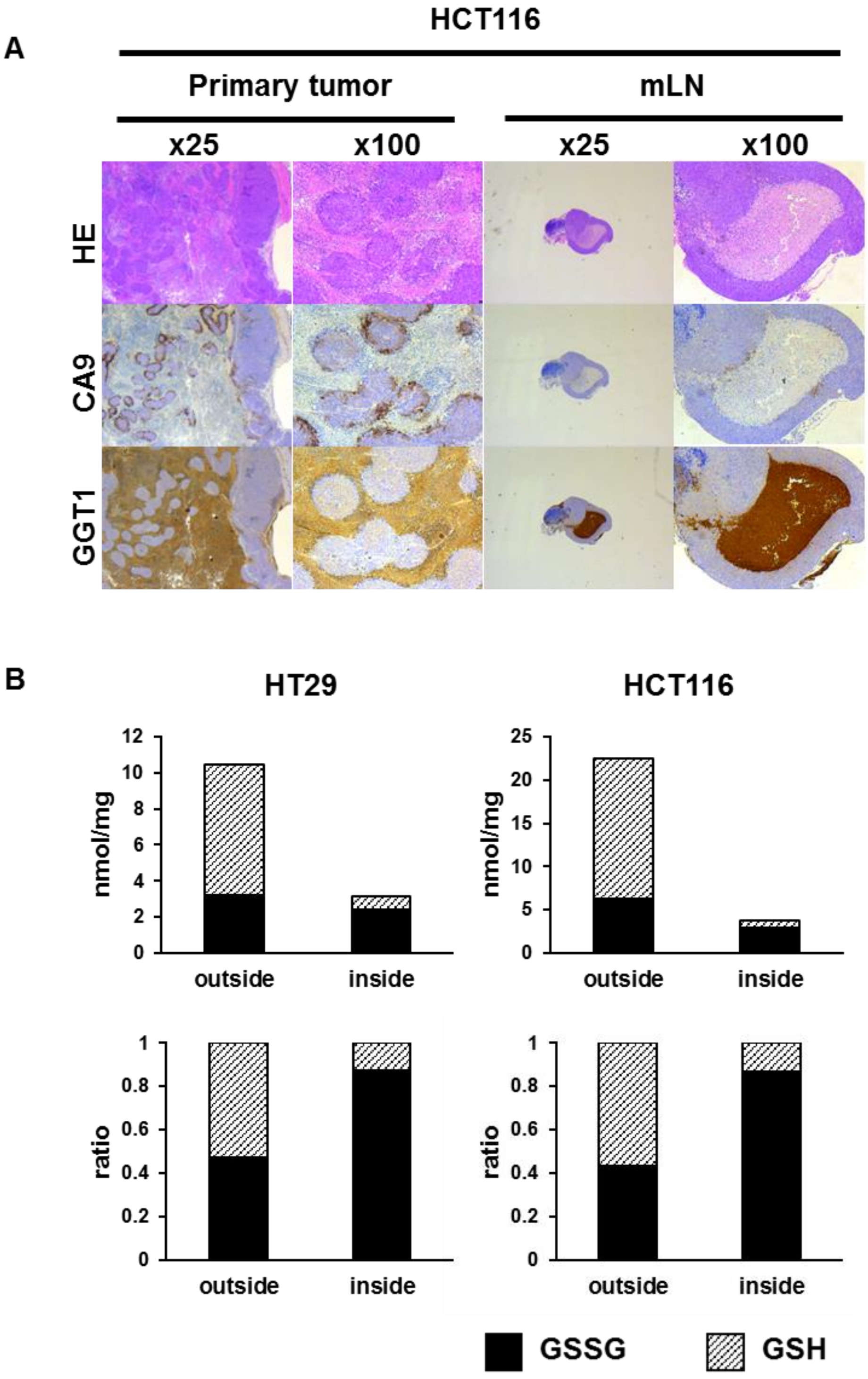

CA9 and GGT1 were expressed inside the primary tumor and in mLNs. GGT1 was accumulated in the area of central necrosis. GSH was depleted inside the tumor.

A) HE staining and immunohistochemistry (CA9 and GGT1) of fixed primary tumor and mLN specimens from orthotopic mouse model of HCT116. **B)** GSH and GSSG concentrations inside and outside the primary tumor (n=3).

Supplementary Figure 5

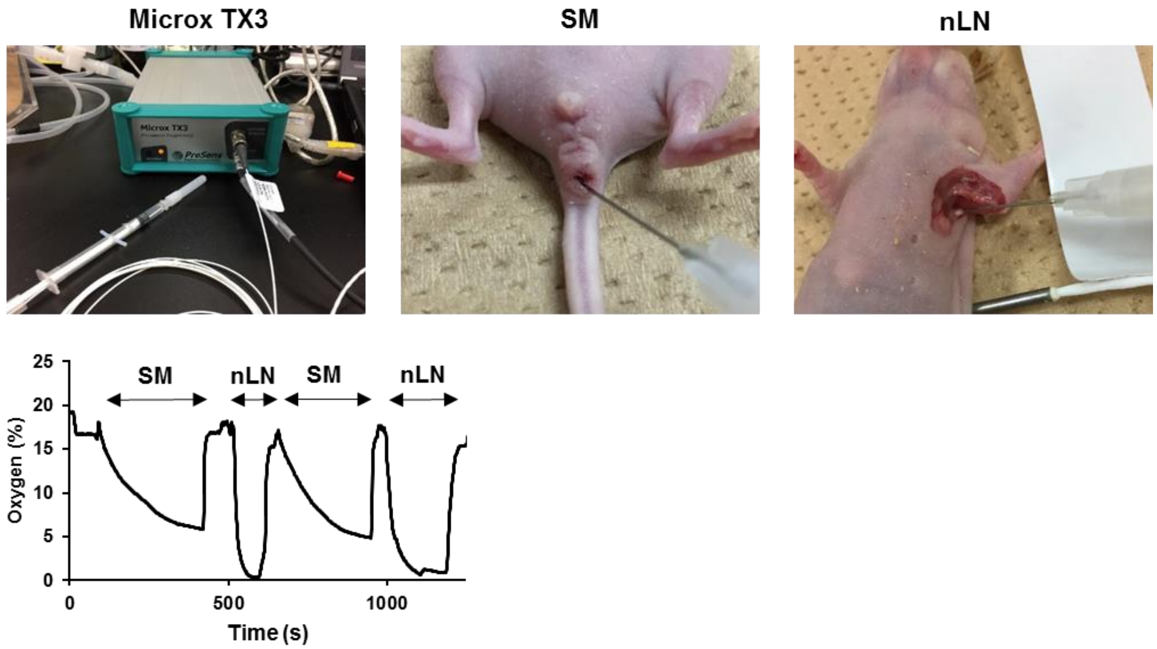

Measurement of tissue oxygen concentrations of rectal submucosa and axillary lymph node (non metastatic lymph node) with Microx TX3. Oxygen concentration of nLN was lower than that of the rectal submucosa. SM, submucosa; nLN, non metastatic lymph node.

Supplementary Figure 6

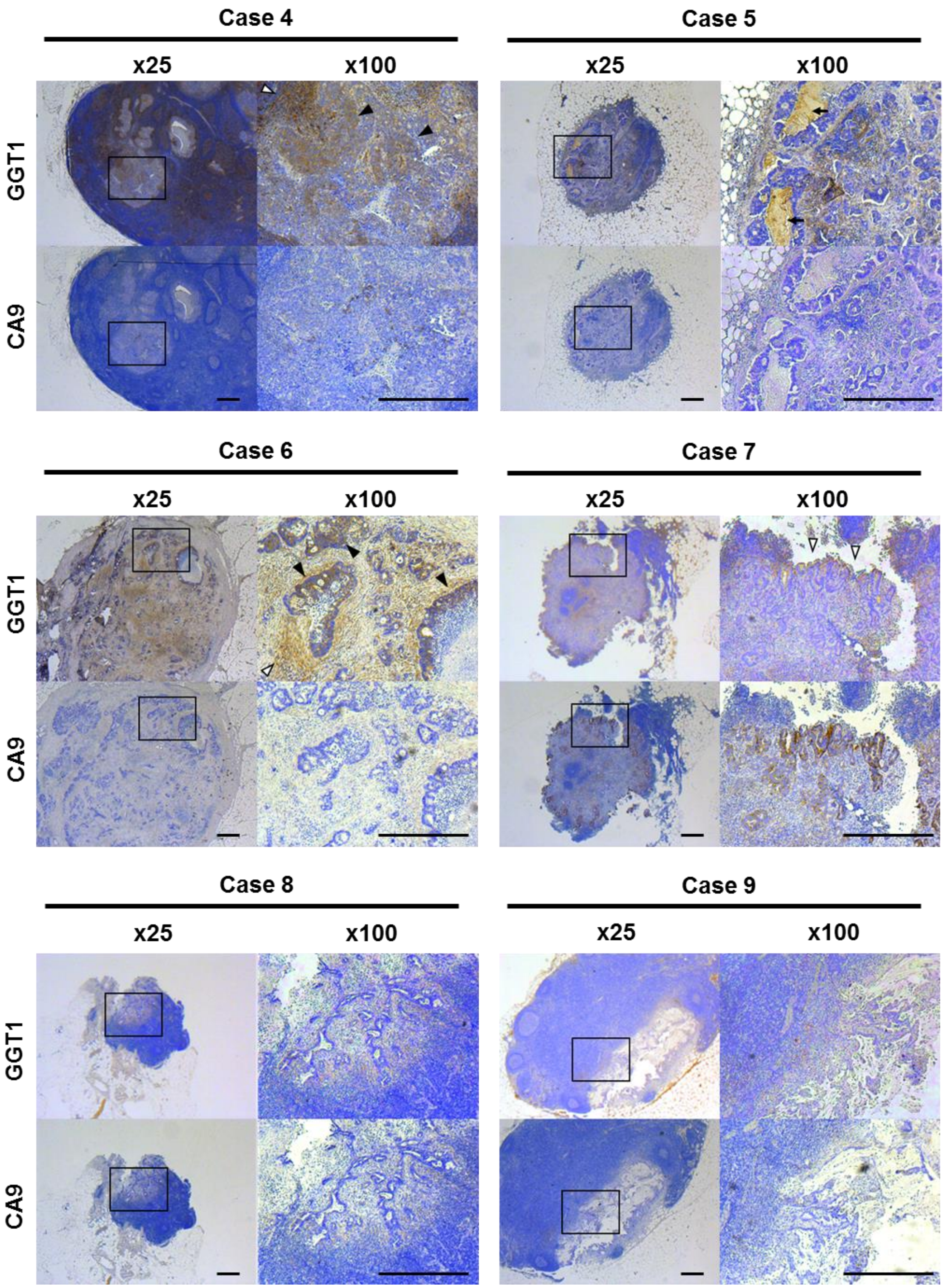

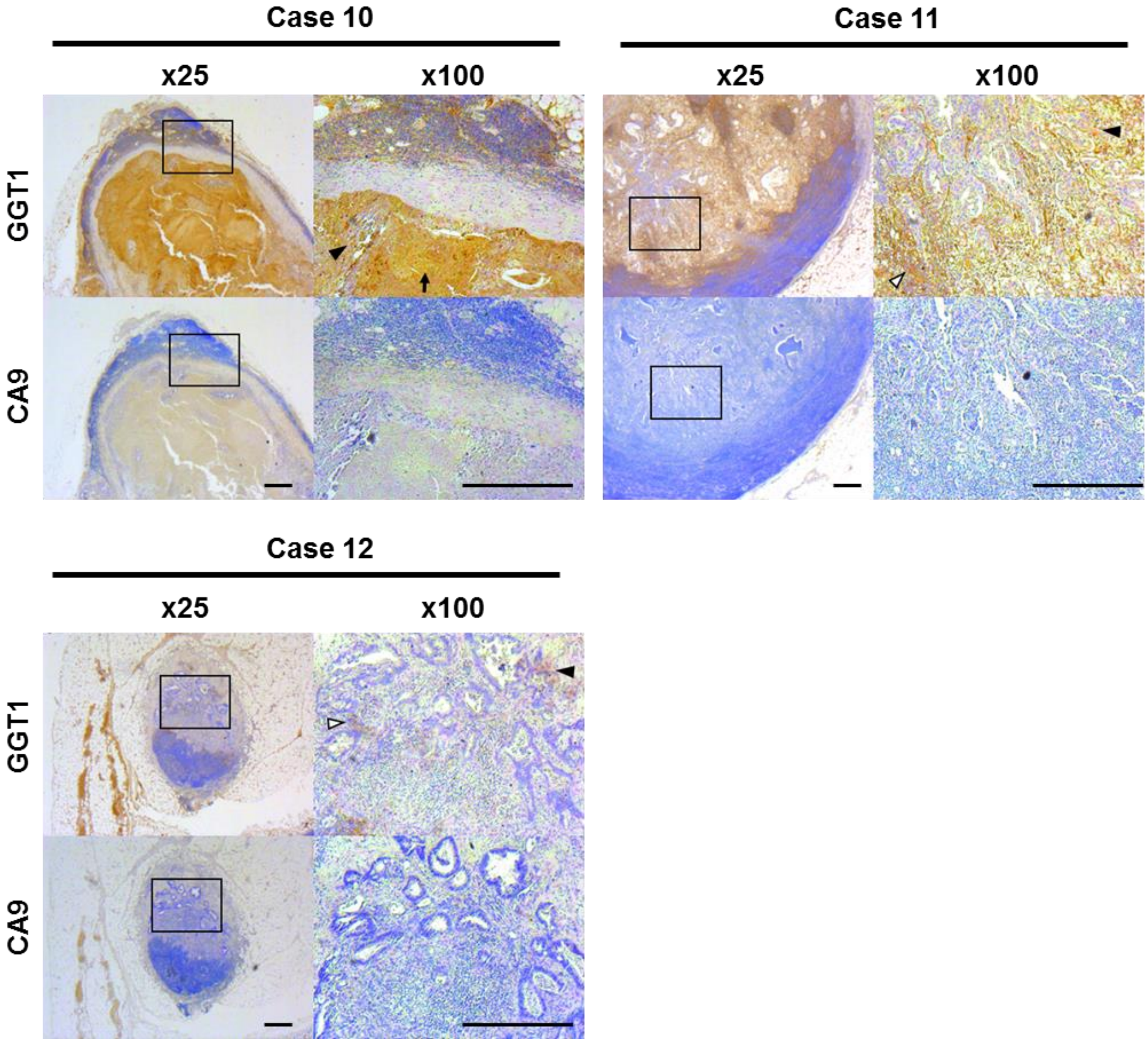

CA9 and GGT1 immunohistochemistry of human mLNs. The mLNs of cases 4-6, 10 and 11 expressed GGT1, while cases 7 and 12 showed only weak expression and cases 8 and 9 expressed little. Scale bar, 500 μm. Black arrowheads: GGT of cancer cells, white arrowheads: GGT surrounding cancer cells, arrows: accumulation of GGT.

Supplementary Figure 7

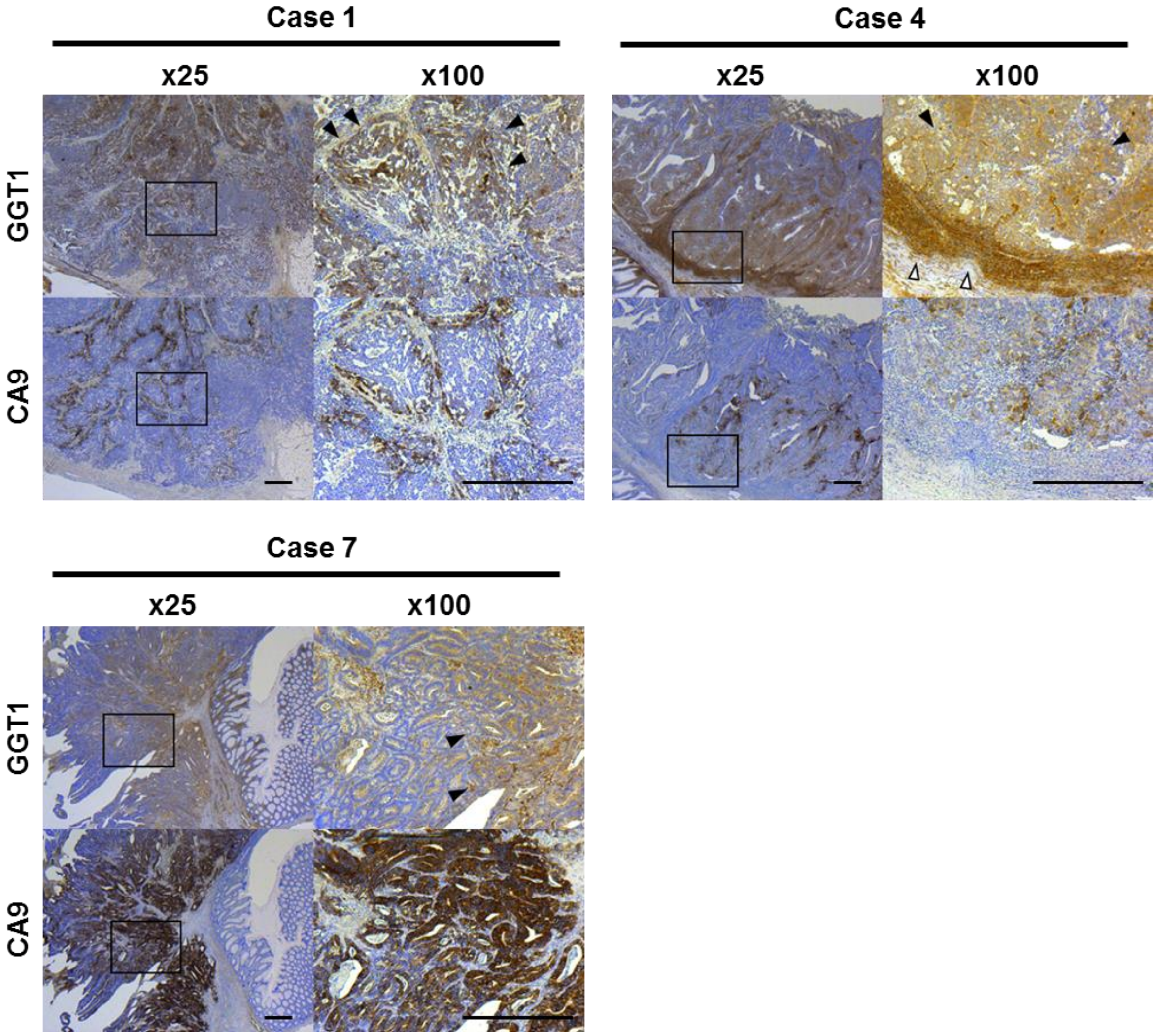

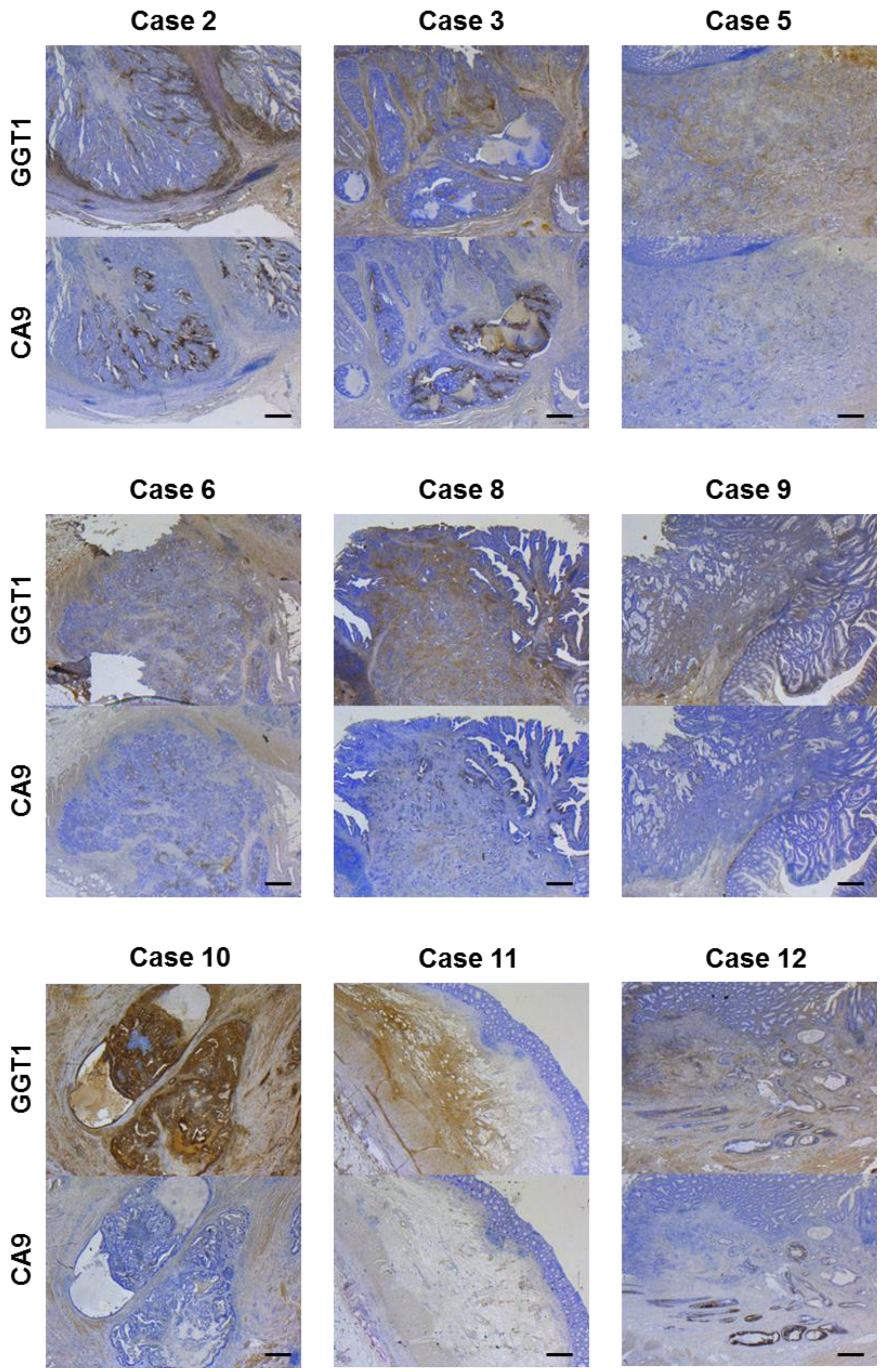

CA9 and GGT1 immunohistochemistry of human primary tumor. In cases 1, 4 and 7, the expression patterns of GGT1 and CA9 were similar, while the expression patterns of

GGT1 and CA9 were different in cases 2, 3, 5, 6 and 8-12. Scale bar, 500 μm. Black arrowheads: GGT of cancer cells, white arrowheads: GGT surrounding cancer cells.
